# Cost-utility and cost-effectiveness analysis of disease-modifying drugs of relapsing–remitting multiple sclerosis: a systematic review

**DOI:** 10.1186/s13561-024-00478-7

**Published:** 2024-02-16

**Authors:** Nasrin Abulhasanbeigi Gallehzan, Majid Khosravi, Khosro Jamebozorgi, Nazanin Mir, Habib Jalilian, Samira Soleimanpour, Saeed Hoseini, Aziz Rezapour, Abbas Eshraghi

**Affiliations:** 1 Health Management and Economics Research Center, Health Management Research Institute, Iran University of Medical Sciences, Tehran, Iran; 2 Faculty of Medicine, Zabol University of Medical Sciences, Zabol, Iran; 3 Department of Health Services Management, School of Health, Ahvaz Jundishapur University of Medical Sciences, Ahvaz, Iran; 4 Department of Medical Library and Information Science, School of Health Management and Information Sciences, Iran University of Medical Sciences, Tehran, Iran; 5 Health Management and Economics Research Center, School of Health Management and Information Sciences, Iran University of Medical Sciences, Tehran, Iran; 6 Student Research Committee, Tabriz University of Medical Sciences, Tabriz, Iran

**Keywords:** Disease-modifying drugs, Relapsing–remitting multiple sclerosis, Cost-utility analysis, Cost-effectiveness analysis

## Abstract

**Background:**

Multiple sclerosis (MS) is a chronic, autoimmune, and inflammatory disease. The economic burden of MS is substantial, and the high cost of Disease-modifying drugs (DMDs) prices are the main drivers of healthcare expenditures. We conducted a systematic review of studies evaluating the cost-utility and cost-effectiveness of DMDs for relapsing–remitting multiple sclerosis (RRMS).

**Materials and method:**

Searches were conducted in PubMed, Web of Science, Scopus, and Embase. The search covered articles published between May 2001 and May 2023. Studies that were written in English and Persian and examined the cost-utility and cost-effectiveness of DMDs in patients with MS were included in our review. Data extraction was guided by the Consolidated Health Economic Evaluation Reporting Standards (CHEERS) checklist, and the quality of economic evaluations was assessed using the Quality of Health Economics Studies Instrument (QHES). All costs were converted to 2020 U.S. dollars using Purchasing Power Parity (PPP).

**Results:**

The search yielded 1589 studies, and 49 studies were eligible for inclusion. The studies were mainly based on a European setting. Most studies employed Markov model to assess the cost–effectiveness. The lowest and highest numerical value of outcome measures were -1,623,918 and 2,297,141.53, respectively. Furthermore, the lowest and highest numerical value of the cost of DMDs of RRMS were $180.67, and $1474840.19, respectively.

**Conclusions:**

Based on the results of all studies, it can be concluded that for the treatment of patients with MS, care-oriented strategies should be preferred to drug strategies. Also, among the drug strategies with different prescribing methods, oral disease-modifying drugs of RRMS should be preferred to injectable drugs and intravenous infusions.

**Supplementary Information:**

The online version contains supplementary material available at 10.1186/s13561-024-00478-7.

## Introduction

MS is a chronic demyelinating disorder of the central nervous system that is clasified as an immune-mediated inflammatory disease [1, 2]. The clinical course and severity of the disease are variable, but the most common symptoms of the disease include paralysis, tingling, weakness, impaired balance and gait, blurred vision or diplopia, vertigo, cognitive impairment, fatigue, and urinary bladder dysfunction [3]. The prevalence of MS has increased in many parts of the world since 2013. The number of patients with MS has increased from 2.3 million in 2013 to 2.8 million in 2020 [4]. A meta-analysis study in 2020 indicated that the annual prevalence of MS had increased by 2.3% in the span of 1985–2018 [5]. The disease usually occurs between 20–50 years of age and women are twice as likely to have MS as men [6].

The course of MS is divided into four types: progressive-relapsing MS (PRMS), RRMS, primary progressive MS (PPMS), and secondary progressive MS (SPMS) [7]. RRMS, the most common form of MS is marked by worsening of neurological symptoms or unpredictable relapses, (also knwn as exacerbations and attacks). A relapse is followed by a remission. During a remission, symptoms partly or completely go away [8]. About 85% of people with MS are initially diagnosed with RRMS, which is characterized by destructive attacks on neurigical function, followed by periods of remission, and without progression of the disease. Approximately 50% of patients with MS will eventually transition to SPMS. This transmission is characterized by progressive worsening of the disease [9]. SPMS affects women twice as often as men [7]. Relapse and disability level are associated with a higher risk for mortality, additional costs, and quality of life (QoL) losses [10].

There are several pharmacological treatments for RRMS. These disease-modifying therapies (DMTs) can reduce the number of relapses, stop or slow the progression of residual disability [10] and delay the progression of the disease but contribute to increased treatment costs [11]. The main goal of different MS treatments is to prevent or delay long-term disabilities. There is currently no definitive cure for MS, but various drugs are being used to control the disease, amongst which are interferon beta and glatiramer acetate, oral drugs (dimethyl fumarate (DMF), teriflunomide and fingolimod), natalizumab and alemtuzumab [12].

MS imposes a substantial economic burden on the healthcare system, patients, caregivers, and society as a whole because of its chronic progressive disease course [13]. The annual healthcare cost per MS patient increased from $ 45,471 in 2011 to $ 62,500 in 2015. In addition, the annual cost of purchasing medication for each MS patient increased from $ 26,772 to $ 43,606 during the same period [14]. The costs of DMDs account for a large proportion of total medical costs (64% to 91%) [15]. A study in Spain indicated that the total cost of MS was € 1395 million per year, with an average annual cost of € 30,050 per patient. In addition to the costs, the disease significantly impacts patients’ QoL, and MS caused a loss of 13,000 quality-adjusted life years (QALYs) annually [16].

A study in France in 2016 estimated the incremental cost-effectiveness ratio (ICER) for delayed-release DMF versus relevant MSDMTs available and demonstrated from both the payer and societal perspectives DMF and IFN beta-1a 44 mcg were the two dominant treatments. IFN beta-1a 30 mcg, IFN beta-1b 250 mcg, teriflunomide, glatiramer acetate, fingolimod were dominated on the efficiency frontier. From the societal perspective, DMF versus IFN beta-1a 44 mcg incurred an incremental cost of €3,684 and an incremental quality-adjusted life year (QALY) of 0.281, corresponding to an ICER of €13,110/QALY [17]. A study in the US demonstrated that over 10 years, peginterferon beta-1a was dominant (i.e., more effective and less costly), with cost-savings of $22,070 and an additional 0.06 QALYs compared with interferon beta-1a 44 mcg and with cost-savings of $19,163 and 0.07 QALYs gained compared with glatiramer acetate 20 mg [18]. A study in 2022 estimated the effectiveness and cost-effectiveness of 360 treatment sequences in RRMS using a microsimulation model from a societal perspective. In this study, the most effective treatment sequence was peginterferon, followed by DMF for patients were at first-line treatment. Patients with relapse or Expanded Disability Status Scale (EDSS) progression on either peginterferon or DMF were then switched to second-line treatment ocrelizumab, then natalizumab, and finally third-line treatment alemtuzumab. This sequence yielded 20.24 ± 1.43 QALYs. Also, the most cost-effective sequence (peginterferon, glatiramer acetate, ocrelizumab, cladribine, and alemtuzumab) yielded 19.59 ± 1.43 QALYs [19].

Given the increasing number of MS patients and available DMTs, and the considerable economic burden associated with MS, it is impoertant to identify which treatment options are most cost-effective. The cost-utility and cost-effectiveness of different oral and injectable DMTs has been evaluated in previous studies, but cost-utility and cost-effectiveness analysis of RRMS treatment sistematically has not yet been put forward in a single study. Starting from this point, we aim to fill the gap in the literature by conducting a systematic review to analyze cost-utility and cost-effectiveness of DMDs for RRMS. For this purpose, the present study aimed to analyze the cost-utility and cost-effectiveness of relapsing–remitting drugs for MS.

## Methods

### Study design

A systematic review was conducted in accordance with the Consolidated Health Economic Evaluation Reporting Standards (CHEERS) 24-item checklist [20].

### Search strategy

We searched Pubmed, Web of Science, Scopus, and Embase databases for eligible studies published until August 2023. The search covered eligible articles published between May 2001 and May 2023. The search of all databases was initially conducted in January 2020 and was updated in August 2023. The search was conducted using combinations of Medical Subject Heading (MeSH) terms for “Disease-Modifying Drugs, Relapsing–Remitting Multiple Sclerosis, Cost-Utility Analysis, Cost-Effectiveness Analysis” to retrieve potentially relevant publications (Additional file 1). Additionally, we searched on Google Scholar based on keywords and examined the reference lists of included articles and grey literatures for additional relevant articles. The search procedure was completed with hand searching.

### Eligibility criteria

The articles included in this review met the Population, Intervention, Comparison, and Outcome (PICOS) criteria contained in WHO guidelines: P: The population comprised patients with MS and taking the drugs for RRMS; I: The intervention comprised DMDs of RRMS; C: The comparison included using other types of drug and treatment methods (if could be substituted); O: Outcomes measure included ICER and costs per natural unit of health measurement; S: Studies employed economic evaluation. In our review, the articles were included if they: (1) published until August 2023 and estimated the cost-utility and cost-effectiveness of DMDs for patients with RRMS. Studies were excluded if they were (1) review, conference abstracts, protocols, letters to the editor, (2) were not published in English and Persian languages, (3) if their full text was not available, and (4) and they did not conduct an original economic evaluation (e.g. effectiveness evaluation, cost evaluation).

### Study selection

After duplicate articles were removed using EndNote software, two reviewers (NAG & MKH) independently reviewed the title and abstract of all articles obtained from the literature searches for eligibility and discussed when discrepancies arose. Next, two reviewers (NAG & MKH) independently evaluated the full-text articles of all identified citations to establish relevance of the article according to the prespecified criteria. In the case of disagreement in the selection process, any discord was resolved by discussion with a third reviewer (NM).

### Data extraction

NM, SS, AE, SH and SS extracted data, and NAG and MKH checked the extracted data. For each study that met the selection criteria, details extracted included the first author's name, year of publication, outcome measure, setting, study population, interventions, type of economic evaluation, perspective, time horizon, willingness to pay (WTP) threshold, discount rate, sensitivity analyses, etc. All costs were converted to 2020 U.S. dollars using Purchasing Power Parity (PPP).

### Quality assessment

Quality assessment was done using the Quality of Health Economics Studies Instrument (QHES). QHES is a validated quality-scoring instrument (score range = 0–100; > 75 = high quality), and a practical quantitative tool which widely used in quality appraisal of cost-effectiveness studies [21]. Using this tool, studies are graded on whether they provide relevant information that is standard to reporting in economic evaluations, such as an explicit statement of the main objective, specify the inclusion and exclusion criteria, the information sources etc. The tool gives weighting scores to different quality indicators (Table 1). In this review the quality scoring was conducted independently by the first and second authors, and then compared for agreement. Disagreements were resolved through subsequent discussions. The agreement on scoring was 77%.

**Table 1:** The quality of health economic studies (QHES) instrument

	**Questions**	**Weight**
1	Was the study objective presented clearly and in a measurable manner?	7
2	Were the perspective of the analysis (health system, third-party payer, etc.) and reason for its selection stated?	4
3	Were variable estimates used in the analysis from the best available source (i.e. randomized control trial—best, expert opinion—worst)?	8
4	If estimates came from a subgroup analysis, were the groups prespecified at the beginning of the study?	1
5	Was uncertainty handled by: (1) statistical analysis to address random events; (2) sensitivity analysis to cover a range of assumptions?	9
6	Was incremental analysis performed between alternatives for resources and costs?	6
7	Was the methodology for data abstraction (including the value of health states and other benefits) stated?	5
8	Did the analytic horizon allow time for all relevant and important outcomes? Were benefits and cost that went beyond 1 year discounted and a justification given for the discount rate?	7
9	Was the measurement of costs appropriate and the methodology for the estimation of quantities and unit costs clearly described?	8
10	Were the primary outcome measure(s) for the economic evaluation clearly stated and were the major short-term, long-term, and negative outcomes included?	6
11	Were the health outcomes measures/scales valid and reliable? If previously tested, valid and reliable measures were not available, was justification given for the measures/scale used?	7
12	Were the economic model (including structure), study methods and analysis, and the components of the numerator and denominator displayed in a clear transparent manner?	8
13	Were the choice of economic model, main assumptions and limitations of the study stated and justified?	7
14	Did the author(s) explicitly discuss direction and magnitude of potential biases?	6
15	Were the conclusion/recommendations of the study justified and based on the study results?	8
16	Was there a statement disclosing the source of funding for the study?	3

## Results

### Study selection

**Fig. 1:**
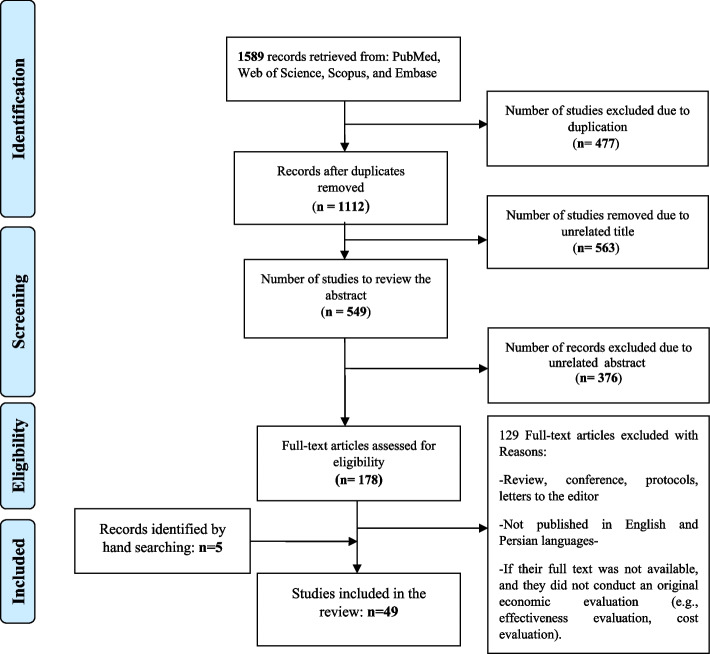
Flow chart of the study selection process

As shown in Fig. 1, the literature search yielded 1589 articles. After the removal of duplicates, titles and abstracts of 549 articles were screened, and 376 irrelevant articles were excluded. Additionally, a further 5 relevant articles were identified by hand searching. A total of 178 articles were selected for full-text evaluation, of which 129 were excluded because they did not meet one or more of the inclusion criteria. Finally, 49 articles met eligibility criteria and were included in our review.

### Characteristics of included studies

Of the included studies, twenty-four studies were carried out in European countries, seven in the United States, four in Canada, six in Iran, four in Saudi Arabia, one in Thailand, one in Colombia, one in Chile, and one in Lebanon. Thirteen studies conducted CEA using a Markov model [18, 22–34], eight studies conducted CUA using a Markov model [35–42], one study conducted CUA using a 31-health-state Markov model [43], one study conducted CEA using a 5-year cohort-based Markov model [44], one study conducted CEA using a 1-year cycle cohort-based Markov state transition model [45], one study conducted CEA using a lifetime Markov model [46], five studies conducted CEA [2, 47–50], two studies conducted both CEA and CBA [10, 51], one study conducted CEA using simulation model [52], one study conducted CEA using a treatment-sequence model [53], one study conducted CUA and budget impact analysis (BIA) using a Markov state transition model [54], one study conducted CEA using a published Markov structure with health states based on the Expanded Disability Status Scale (EDSS) [55], one study conducted CEA using a Markov state transition model [56], one study conducted CEA using a Markov economic model [57], five studies conducted CEA using a cohort Markov economic model [10, 13, 17, 51, 58], one study conducted CEA using a microsimulation model [19], one study conducted CEA using a discrete-time Markov model [59], one study condcuted CEA using a cohort-based multi-state Markov model [60], and one study conducted CEA using a probabilistic Markov model (second-order Monte Carlo simulation) [61] (Table 2).

**Table 2:** Characteristics of studies included in the review

First author’s name (Year)	Costing year	Setting	Population	Compared interventions	Type of economic evaluation	Perspective	Time horizon	WTP Threshold	Discount rate	Sensitivity analyses	Quality index decision based on % score	Sponsor
Smets et al. (2023) [53]	2022	Netherlands	1000 pwMS	Ocrelizumab/ ofatumumab compared with eight other drug classes	Health outcomes (i.e., lifetime relapses, time to Expanded Disability Status Scale [EDSS] 6), lifetime quality-adjusted life years [QALYs]) and cost-effectiveness (i.e., net health benefit [NHB]) (the ErasmusMC/iMTA MS)	Health-economic and societal perspective	Lifetime	Monetary value of a QALY (€50,000 for MS in The Netherlands) / willingness-to-pay’ of €50,000 per QALY	Costs: 4%, effects 1.5%	Probabilistic analysis	High	Dutch National MS Foundation, Merck for MS-related research & Merck for MS-related research
Matni et al. (2022) [54]	2019	Lebanon	HDA -RMS patients	Assessing cost-utility and financial impact of cladribine tablets in HDA -RMS patients compared with other HDA-RMS therapies	CUA & budget impact model (A Markov state transition model)	Lebanese National Social Security Fund (NSSF) perspective	50-year	The willingness to pay threshold of 22,000 USD (approximately three times the gross domestic product [GDP] per capita) per QALY	3.5%	Deterministic sensitivity analysis, Probabilistic sensitivity analysis	High	Merck Serono Middle East FZ-Ltd, an affiliate of Merck KGaA, Darmstadt, Germany
Spelman et al. (2022) [55]	2019	UK	3935 Adults with highly active RRMS (HA-RRMS) with inadequate response to BRACETD (first line therapies)	The comparative effectiveness of switching to natalizumab or fingolimod or within BRACETD	CEA (published Markov structure with health states based on the Expanded Disability Status Scale)	UK third-partypayer perspective		Willingness-to-pay threshold of £30,000 per quality-adjusted life-year (QALY) gained	3.5%	Three-way-multinomial-propensity-score–matched analysis	High	Biogen International GmbH (Baar, Switzerland); MSBase receives general financial support from Biogen, Genzyme, Merck (MSD), Merck Serono, Novartis, Roche, and Teva
Espinoza et al. (2021) [38]	2018	Chile	261 Patients with HAD-RRMS	Cladribine compared with alemtuzumab, natalizumab, and ocrelizumab	CEA (Markov model)	Chilean health care public sector	45 years	Equivalent to 3 GDP per capita	3%	Deterministic & probabilistic sensitivity analysis	High	Merck S.A., Chile (a business of Merck KGaA, Darmstadt, Germany), commissioned to Pontificia Universidad Católica (Santiago, Chile)
Bohlega et al. (2021) [56]	-	Kingdom of Saudi Arabia	Patients with high disease activity compared with other HDA-RRMS therapies	Cladribine tablets versus other DMDs (alemtuzumab, dimethyl fumarate, fingolimod, interferon beta-1a (subcutaneous and intramuscular) and beta-1b, natalizumab, and teriflunomide) in the treatment of HDA-RRMS	CEA (Markov state transition model),	Kingdom of Saudi Arabia payer’s perspective	50-year	The willingness-to-pay threshold of Saudi Riyal (SAR) 225 326 (approximately 3 times of gross domestic product per capita) per QALYs gained	3.5%	One-way & probabilistic Sensitivity analysis	High	Merck Serono Middle East FZ-Ltd, an affiliate of Merck KGaA, Darmstadt, Germany
Ayati et al. (2021) [43]	2019	Iran	Iranian patients with relapsing multiple sclerosis	Ocrelizumab in comparison to natalizumab	CUA (A 31-health-state Markov model)	Societal perspective	10 years	Iran’s pharmacoeconomic WTP threshold ($2709)	Costs: 7.2% and QALYs: 3.5%	Deterministic sensitivity analysis and probabilistic sensitivity analysis	High	Roche Corporation, Roche Pars Ltd
Ayati et al. (2021) [44]	2020	Iran	Patients with HDA-RMS as on and off-treatment	Cladribine tablets compared to natalizumab	CEA (A 5-year cohort-based Markov model)	Societal perspective	5 years	WTP threshold of 1 to 3 gross domestic product (GDP) per capita	3.5%	Deterministic sensitivity analysis and probabilistic sensitivity analysis	High	None
Lasalvia et al. (2020) [57]	2016	Colombia	Highly active RRMS patients	Natalizumab Compared With Fingolimod	CEA (Markov economic model)	Colombian healthcare system perspective	5 years	3 times the gross domestic product per capita of Colombia, equivalent to $17 401	5%	Univariate and probabilistic sensitivity analysis	High	Stendhal
Poveda et al. (2019) [39]	2018	Spain	Patients with RMS with high disease activity	Cladribine Tablets compared with fingolimod	CEA (Markov model)	National Health System	50 years	-	3%	Deterministic and probabilistic sensitivity analyzes	High	Merck, S.L.U., an affiliate of Merck KGaA Darmstadt, Germany
Dembek et al. (2014) [27]	2010	Spain	1,000 RRMS patients	Injectable DMTs (interferon beta-1a (SC IFNb-1a), interferon beta-1b (IFNb-1b) and glatiramer acetate (GA)) for the first-line treatment	CEA (Markov model)	Societal perspective	30 years	-	3%	Univariate and probabilistic sensitivity analyses	High	Biogen Idec
Ginestal et al. (2023) [61]	Unit costs: 2021, Cost of the drugs: 2022	Spain	RRMS patients	Cost–effectiveness of cladribine tablets and dimethyl fumarate	CEA (probabilistic Markov model (second-order Monte Carlo simulation))	Spanish National Health System perspective	10 years	WTP of 25,000€ per QALY gained	3%	Univariate sensitivity analyses	High	Merck, S.L.U., Madrid, Spain
Furneri et al. (2019) [28]	2015 (in Euro)	Italy	RRMS patients	Early escalation to natalizumab vs. switching among immunomodulators, followed by late escalation to natalizumab, in patients affected by RRMS	CEA (Markov model)	Italian societal perspective	Over a 50 year	Willingness to pay threshold of € 50,000 per QALY gained	3.50%	Univariate deterministic and probabilistic sensitivity analyses	High	Biogen Italy (Milan, Italy)
Cortesi et al. (2022) [60]	2020	Italy	Patients with secondary progressive multiple sclerosis (SPMS)	The siponimod cost-effectiveness profile and its relative budget impact compared with other DMTs,	CEA (A cohort-based multi-state Markov model)	Italian National Healthcare System perspective	Life-time horizonand 1-year cycle	WTP of €40,000 per QALY gained	3%	One-way sensitivity analysis and probabilistic sensitivity analysis	High	Novartis SpA
Stanisic et al. (2019) [34]	2017	Italy	Patients with RRMS	Alemtuzumab in comparison with subcutaneous IFN β-1a, natalizumab and fingolimod	CEA (Markov model)	Payer perspective	Lifetime horizon (i.e 50 years)	WTP threshold in Italy (€40,000/QALY)	3.5%	Deterministic one-way sensitivity analysis and probabilistic sensitivity analyses	High	Sanofi SpA
Montgomery et al. (2022) [58]	2020	UK	Patients with active secondary progressive multiple sclerosis	Oral siponimod versus continued oral or infused relapsing–remitting multiple sclerosis disease-modifying therapies	CEA (cohort Markov model)	UK National Health Service perspective	1 year	WTP threshold of £30,000/QALY	3.5%	Probabilistic and deterministic sensitivity analyses	High	Novartis Pharmaceuticals UK Ltd
Rezaee et al. (2022) [29]	2019	Iran	120 patients with RRMS	Rituximab against natalizumab	CEA (Markov model)	Societal perspective	Over 1 year	$ 37,641 (3* GDP)	Costs = 5.8 & Outcomes = 3%	One-way sensitivity analysis and Probabilistic Sensitivity Analysis	High	Shiraz University of Medical Sciences
Becker et al. (2011) [47]	2009	US	Patients with RRMS	The impact of selecting the 2-year cohort rather than the all-patient cohort for IM IFNβ-1a on the results of the original model	CEA	Health care payer perspective	2 years	-	-	Sensitivity analysis	High	Biogen Idec Inc
Kantor (2023) [48]	2020	US	Patients with RRMS	Ozanimod compared with teriflunomide, interferon beta-1a, interferon beta-1b, glatiramer acetate, fingolimod, and dimethyl fumarate	CEA	-	Over 1 year	-	-	Sensitivity analysis	Fair	Bristol Myers Squibb
Baharnoori et al. (2022) [13]	2021	Canada	Adults with RRMS	Ofatumumab	CEA (Markov cohort model)	Canadian healthcare system perspective	25 years	WTP threshold of $50,000 Canadian dollars (CAD) per QALY gained	1.5%	Probabilistic sensitivity analysis	High	Novartis Pharmaceutical Canada Inc
Lazzaro et al. (2022) [40]	2019	Italy	RRMS naïve and 1000 RRMS experienced patients	To compare the costs and QALYs of teriflunomide in RRMS naïve patients vs. RRMS patients previously treated (experienced) with other DMTs (alemtuzumab; cladribine; fingolimod; natalizumab; ocrelizumab)	CUA (A four health states Markov model-supported cost-utility analysis)	Healthcare sector & societal perspective	7 years	WTP = 0 per incremental QALY gained	3%	One-way, scenario and probabilistic sensitivity analyses	High	Sanofi S.r.l
Pinheiro et al. (2020) [45]	2016	Portugal	Patients with highly active RRMS	Cost-utility of cladribine tablets versus fingolimod	CEA & CUA (A 1-year cycle cohort-based Markov state transition model)	Payers’ perspective	50 years	-	5%	Probabilistic and deterministic sensitivity analyses	High	Merck S.A
Martins et al. (2023) [33]	2018	Portugal	Treatment-naïve RMS, previously treated RMS, and PPMS patients	The clinical and econoemmic impact of ocrelizumab relative to current clinical practice, including interferon β-1a, dimethyl fumarate, glatiramer acetate, teriflunomide, fingolimod, and natalizumab	CEA (Markov model)	Societal perspective	A lifetime time-horizon with annual cycles	-For the RMS population: WTP greater or equal to €18,000/QALY -For the PPMS population: WTP values higher than €81,000/QALY	5%	Scenario analysis and probabilistic sensitivity analysis	High	Roche Farmaceutica e Quimica, Lda., Portugal
AlRuthia et al. (2021) [49]	-	Saudi Arabia	146 patients with RRMS	Comparing the cost-effectiveness of orally administered medications (e.g., fingolimod, dimethyl fumarate, and teriflunomide), interferon (IFN)-based therapy, and monoclonal antibodies (MABs) (e.g., natalizumab and rituximab)	CEA	Public healthcare payer perspective	At least 1 year	-	-	-	Poor	King Saud University, Riyadh, Saudi Arabia
Versteegh et al. (2022) [19]	2019	Netherlands	382 Dutch patients with MS	Effectiveness and Cost-Effectiveness of 360 DMTES in MS	CEA (microsimulation model)	Societal perspective	Lifetime	€50 000 per QALY	Effects: 1.5% & costs: 4%	Probabilistic sensitivity analyses	High	The Erasmus University Medical Center
Nakhaipour et al. (2020) [59]	2018	Canada	Patients aged 10 years and above with RMS	The incremental cost-effectiveness of fingolimod versus IFN β-1a	CEA (discrete-time Markov model)	Canadian health care system	2 years	WTP threshold of Canadian dollars (CAD) 50,000 per quality-adjusted life-year	1.5%	One-way sensitivity analysis and probabilistic sensitivity analysis	High	Novartis Pharmaceuticals Canada Inc., Dorval, Quebec, Canada
Schur et al. (2021) [30]	2020	Switzerland	Adult patients with secondary progressive multiple sclerosis (SPMS) with active disease	The cost effectiveness and budget impact of siponimod compared to interferon beta-1a	CEA (Markov model)	Swiss health insurance perspective	A cycle length of 1 year and life-long time horizon	A WTP threshold of CHF 100,000 per QALY gained	3%	One-way deterministic and probabilistic sensitivity analyses	High	Novartis Pharma Schweiz AG
Alharbi et al. (2023) [62]	2022	Saudi Arabia	93 RRMS	Comparing the direct medical cost and consequences between rituximab and natalizumab in managing RRMS, and exploring the cost and consequence of ocrelizumab in managing RRMS as a second-choice treatment	-	Public healthcare institutions	6 months	-	-	-	Fair	King Saud University, Riyadh, Saudi Arabia
Gani et al. (2008) [22]	2005	UK	2048 MS patients	Natalizumab compared with interferon-β, glatiramer acetate and best supportive care	CEA (Markov model)	UK societal cost perspective	30 years	£36,000 per QALY	3.5%	Univariate sensitivity analysis	High	Biogen Idec Ltd
Chilcott, et al. (2003) [2]	-	UK	Patients with RRMS and SPMS	Four disease-modifying treatments: interferon betas, glatiramer acetate for relapsing–remitting and interferon betas, glatiramer acetate for secondary progressive multiple	CEA	UK National Health Service	20 years	£20 000	Discounted costs at 6% per annum, the discounted quality of life benefits at 1.5% per annum	Multivariate Monte Carlo sensitivity analysis	High	National Institute for Clinical Excellence
Chevalier et al., (2016) [17]	2015	France	1,000 patients	Glatiramer acetate, IFNbeta-1a 30mcg intramuscularly and 44mcg subcutaneously, IFN beta-1b 250mcg and teriflunomide as first-line therapies and fingolimod and natalizumab, as second-line therapies	CEA (cohort-based Markov model)	Payer and societal	30 years	-	4% per annum during the first 30 years and2% after as requested by the French guidelines	Univariate and probabilistic	High	Biogen France SAS
Chanatittarat, et al. (2018) [35]	2016	Thailand	105 MS patients (mean age 37.8 years)	Best supportive care (BSC), fingolimod, IFNβ − 1b, and IFNβ − 1a	CUA (Markov model)	Societal	Month cycle length, lifetime horizon or 30 years	WTP threshold of USD 4,500 per QALY gained	3 percent, Costs were converted to USD using 2016 average annual exchange rate of 35.26 Thai baht (THB) per 1 USD	Univariate and probabilistic	High	None
Brown, et al. (2000) [52]	1999	Canada/ Nova Scotia	1,000 females and 1,000 males followed 40 years	interferon beta-1b (IFN¯-1b)	CEA (simulation model)	Ministry of health (MOH)	Unspecified lifespan	-	5%	Sensitivity analysis (the cumulative probabilities)	High	Canadian Coordinating Office for Health Technology Assessment (CCOHTA)
Bozkaya, et al. (2017) [23]	2016	USA	for relapsing–remitting multiple sclerosis (RRMS)	natalizumab (NTZ), dimethyl fumarate (DMF), and peginterferon beta-1a (PEG) with fingolimod (FIN), glatiramer acetate (GA, 20 mg daily), and subcutaneous interferon beta-1a (IFN, 44 mcg),	CEA (Markov Model)	Third-party payer	Three-month cycles were modeled over a 10-year time horizon	-	3%	One-way deterministic sensitivity analysis	High	Biogen
Alsaqa’aby, et al. (2017) [24]	2015	Saudi Arabia (Tertiary care hospital)	1000 RRMS patients (for more than 400real MS patients)	Oral agents v (fingolimod, teriflunomide, dimethyl fumarate,) vs. interferon (IFN)-b1	CEA: Cohort Simulation Model (Markov Model(	Saudi Payer	20 years and an annual cycle length	$100,000	3%, All costs were reported in Saudi Riyals (SAR) and converted into the equivalent value of 2015 US dollars	One-way and probabilistic (A probabilistic sensitivity analysis based on a second-order Monte Carlo simulation (1000 times))	High	None
Hernandez, et al. (2016) [18]	2014	USA	RRMS and includes adult patients. The population is 29.2% male with a mean age of 36.5 years	Peginterferon beta-1a compared with interferon beta-1a and glatiramer acetate	CEA( Markov cohort model)	US payer	over 10 years	$50,000	3%	Probabilistic sensitivity analysis	High	Biogen
Hernandez, et al. (2017) [25]	2015	Scotland	RRMS	Peginterferon beta-1a and Interferon beta-1a 30 mcg and Interferon beta-1a 22 mcg and Interferon beta-1a 44 mcg and Interferon beta-1b and Glatiramer acetate 20 mg	CEA (Markov cohort model)	National Health Service and Personal Social Services	over 30 years	£20,000 per QALY	and discounted at 3.5% per year	Probabilistic sensitivity analysis	High	Biogen
Sawad, et al. (2017) [63]	2014	USA	patients with RRMS Healthcare costs data were obtained from a study conducted in 2004 by Kobelt et al. assessing the cost of MS disease by stratified EDSS health states	Strategy 1: (symptom management [SM] alone), vs. Strategy 2: (SM and IFN-β-1a), vs. Strategy 3:(SM and natalizumab) vs. Strategy 4: (SM and alemtuzumab)	CEA (Markov model)	Third-party payer	Over 20 years	$100,000 WTP threshold per QALY	1- All costs were inflated to 2014 US$ by using the US\ 2- costs were discounted using an annual discount rate of 3%	One-way, Probabilistic sensitivity analysis (second-order Monte Carlo simulation	High	None
Hashemi-Meshkini A, et al. (2018) [26]	2016	Iran	1,000 patients with relapsing–remitting MS (RRMS)	Pegylated versus non-pegylated interferon beta 1a	CEA (Markov model)	payer perspective (patients and third-party payers)	One-month cycles over 10 years	15,945 USD	Cost discount rate (5%), Utility discount rate (3%)	One-way deterministic sensitivity analysis	High	None
Michels, et al. (2019) [32]	2016–2017	Netherlands	Derived from a meta-analysis study (113 for cladribine group)	Cladribine tablets vs. alemtuzumab and fingolimod	CEA	Societal	Unspecified lifespan	€50,000/QALY gained	4% for costs and 1.5% for outcomes	Deterministic and probabilistic sensitivity analysis	High	Merck B. V group
Imani, et al. (2012) [36]	2011	Iran	Model-based- population is not clear	Symptom Management vs. Avonex, Betaferon, Rebif, CinnoVex	CUA (Markov model)	Healthcare	Unspecified lifespan	US$50,000/QALY gained	7.2% annually	Sensitivity analysis	High	Tabriz University of Medical Sciences
Janković, et al. (2009) [31]	2009	Serbia	Model-based—the population is not clear	Symptom management alone vs combination with subcutaneous glatiramer acetate (SC GA), subcutaneous interferon β-1a (SC IFNβ-1a), intramuscular interferon β-1a (IM IFNβ-1a), or subcutaneous interferon β-1b (SC IFNβ-1b)	CEA	Societal	lifetime (40 years)	WTP 5,000,000.00 RSD	3% annually	Multiple univariate sensitivity	High	Serbian Ministry of Science and Ecology
Maruszczak, et al. (2015) [41]	2013–2014	UK	Derived from a systematic review- the population is not clear	fingolimod vs. dimethyl fumarate (DMF)	CUA (cohort Markov model)	NHS and Personal Social Services	Lifetime (50 years)	£20,000 and £30,000/QALY	3.5% for both costs and benefits	Deterministic & Probabilistic sensitivity analysis	High	Novartis Pharmaceuticals UK Ltd, Camberley, UK
16.Mantovan et al. (2019) [42]	Euros inflated to June 2018	Italy	Cohort and RCT based, the number of 1237 patients	Dimethyl fumarate vs. other first-line alternatives	CEA (Markov model)	Societal	Lifetime (50 years)	€ 50,000 per QALY gained	3.5% for both costs and outcomes	Univariate deterministic and multivariate probabilistic	High	Biogen Italia (Milan, Italy)
Najafi, et al. (2015) [50]	2012	Iran	140 patients	Avonex vs. CinnoVex	CEA	Ministry of Health and Medical Education	1 year	Not clear enough	Not used	Two-way sensitivity analysis	High	Iran University of Medical Sciences (IUMS)
Nuijten, et al. (2002) [46]	1998	UK	The number of 560, 372, & 358 patients based on the previous three RCTs	Preventive treatment with interferon beta, No preventive treatment	CEA & CUA (lifetime Markov process model)	Third-party payer & Societal	Lifetime (25 years)	-	6% annually	Univariate sensitivity analyses	High	None
Soini, et al. (2017) [10]	2014	Finland	713 patients	DMF 240 mg PO BID, teriflunomide 14 mg once daily, GA 20 mg SC once daily, IFN-β1a 44 mg SC TIW, IFN-β1b 250 mg SC EOD, IFN-β1a 30 mg IM QW, best supportive care (BSC)- placebo	CEA/CBA (cohort Markov model)	Finnish payer perspective and Scenario analysis with a societal perspective	15 years	€ 68,000 per QALY gained	3% annually	Probabilistic sensitivity analysis	High	Sanofi Genzyme
Su, et al. (2016) [64]	2013 Canadian dollars	Canada	Cohort and trial based 308 patients	DMF, Glatiramer Acetate (GA), Rebif (Interferon-b 1a SC) 44 mcg	CEA (Markov cohort model)	Ministry of Health	Lifetime (20 years)	Canada ($50 000–60 000)	5% for both health and economic outcomes	One-way and probabilistic sensitivity analyses	High	Biogen
Zhang, et al. (2014) [51]	Inflated to 2012 dollars	USA	A cohort of 1,000 patients	Fingolimod, Teriflunomide, dimethyl fumarate, intramuscular (IM) interferon (IFN)-b1a	CBA/CEA (Markov model)	Societal	5 years	US$ 150,000 per (QALY)	3% annually	One-way and probabilistic sensitivity analysis	High	None
Zimmermann et al., (2018) [37]	2017	US	Treatment-naïve adults with RRMS or PPMS	DMTs for RRMS (first-line: dimethyl fumarate, glatiramer acetate, interferon β-1a, interferon β-1b, peginterferon β-1a, teriflunomide, natalizumab, fingolimod, and ocrelizumab; second-line: alemtuzumab, natalizumab, fingolimod, and ocrelizumab), ocrelizumab for PPMS, and supportive care	CUA ( Markov model)	US payer perspective	One year	$150,000	3% annually	One-way and probabilistic sensitivity analyses	High	The Institute for Clinical and Economic Review

One study was conducted from the UK societal cost perspective [22], two from UK National Health Service [2, 58], one from UK third-party payer perspective [55], two from Canadian healthcare system perspective [13, 59], one from Chilean health care public sector perspective [38], one from Kingdom of Saudi Arabia payer’s perspective [56], two from Payer perspective [34, 45], one from the Colombian healthcare system perspective [57], one from Italian societal perspective [28], one from Italian National Healthcare System perspective [60], one study from Swiss health insurance perspective [30], one from Payer and societal perspective [17], eleven studies from societal perspective [19, 27, 29, 31–33, 35, 42–44, 51], three studies from Ministry of Health perspective [50, 52, 64], one from Third-party payer perspective [23], one from Saudi payer perspective [24], two from US payer perspective [18, 37], one from US health care payer perspective [47], one from both National Health Service and Personal Social Services perspective [25], two from Spanish National Health System [39, 61], one from third-party payer perspective [63], one from both patients and third-party payers perspective [26], one from healthcare perspective [36], one from public healthcare perspective [49], one from both National Health Service and Personal Social Services perspective [41], one from both third-party payer & Societal [46], and one from Finnish payer perspective and Scenario analysis with a societal perspective [10], one from both health economics and societal perspective [53], one from Lebanese National Social Security Fund (NSSF) perspective [54], and one from both healthcare sector & societal perspective [40].

Twenty-seven studies were conducted under the sponsorship of a pharmaceutical/biotechnoloy company [10, 13, 17, 18, 22, 23, 25, 27, 28, 30, 33, 34, 38, 39, 41, 42, 47, 48, 53–56, 58–61, 64]. Seven studies had no sponsorship [24, 26, 35, 44, 46, 51, 63]. The time horizon was variable; in some articles, it was between 5–10 or over [18, 23, 26, 51] years, while in others, it was 50 years or over [28, 39, 41, 42, 54, 56]. Discount rates were very similar, mostly between 3% and 5–6%. For more details, see Table 2.

Sensitivity analyses were done in the majority of the studies. Sensitivity analyses methods varied with two studies using one-way deterministic sensitivity analysis [23, 26], nine studies using one-way and probabilistic sensitivity analyses [24, 29, 37, 51, 56, 59, 60, 63, 64], one study using one-way, scenario and probabilistic sensitivity analyses [40], two studies using one-way deterministic and probabilistic sensitivity analyses [30, 34], seven studies using probabilistic sensitivity analysis [10, 13, 18, 19, 25, 48, 53], three studies using univariate sensitivity analysis [22, 46, 61], one study using Multivariate Monte Carlo sensitivity analysis [2], three study using sensitivity analysis [36, 47, 52], one study using Multiple univariate sensitivity analysis [31], four studies using both univariate and probabilistic sensitivity analysis [17, 27, 35, 57], one using univariate deterministic and probabilistic sensitivity analyses [28], nine studies using both deterministic & probabilistic sensitivity analysis [32, 38, 39, 41, 43–45, 54, 58], one study using both univariate deterministic and multivariate probabilistic [42], one study using two-way sensitivity analysis [50], one study using three-way-multinomial-propensity-score–matched analysis [55], and one study using scenario and probabilistic sensitivity analyses [33] (Table 2).

Eight studies analyzed the injectable DMDs of RRMS [2, 18, 25, 26, 46, 47, 50, 52], in three studies symptom management [31, 36] and supportive care [27] were included in the cost-effectiveness analysis in addition to injectable form of medication; three studies analyzed the oral DMDs of RRMS [39, 41, 45], eleven studies analyzed both injectable and oral DMDs for RRMS [10, 17, 24, 30, 35, 42, 48, 51, 59, 60, 64]. In three studies, in addition to these two forms of medications, the best supportive care (BSC) strategy [10, 30] and symptom management [24] were included in cost-effectiveness analysis. Eight studies analyzed both oral and intravenous infusions DMDs of RRMS [32, 38, 40, 44, 54, 55, 57, 58]. Ten studies analyzed all three types of DMDs of RRMS [13, 19, 23, 33, 34, 37, 49, 53, 56, 64]. In four studies, in addition to these three forms of medication, the BSC strategy was included in cost-effectiveness analysis [13, 33, 34, 37]. and three studies analyzed the injectable and intravenous infusions DMDs for RRMS [22, 28, 63]. In two studies, symptom management [63] and BSC [22] strategies were analyzed in addition to these two forms of medications. Three study analyzed only intravenous infusions DMDs for RMS [29, 43, 61, 62] (Table 2).

### Quality of included studies

The studies included in the literature review were of variable quality (Table 2). Forty-four studies were graded high, 2 were thought to be fair and 1 was poor.

The proportion of studies that met the criteria for reporting of economic evaluations used in the quality index tool is shown in Table 3.

**Table 3:** Proportion of studies that met the selected criteria for grading economic evaluations

	**Questions**	**N**
1	Was the study objective presented clearly and in a measurable manner?	49
2	Were the perspective of the analysis (health system, third-party payer, etc.) and reason for its selection stated?	48
3	Were variable estimates used in the analysis from the best available source (i.e. randomized control trial—best, expert opinion—worst)?	37
4	If estimates came from a subgroup analysis, were the groups prespecified at the beginning of the study?	49
5	Was uncertainty handled by: (1) statistical analysis to address random events; (2) sensitivity analysis to cover a range of assumptions?	47
6	Was incremental analysis performed between alternatives for resources and costs?	43
7	Was the methodology for data abstraction (including the value of health states and other benefits) stated?	45
8	Did the analytic horizon allow time for all relevant and important outcomes? Were benefits and cost that went beyond 1 year discounted and a justification given for the discount rate?	46
9	Was the measurement of costs appropriate and the methodology for the estimation of quantities and unit costs clearly described?	43
10	Were the primary outcome measure(s) for the economic evaluation clearly stated and were the major short-term, long-term, and negative outcomes included?	47
11	Were the health outcomes measures/scales valid and reliable? If previously tested, valid and reliable measures were not available, was justification given for the measures/scale used?	49
12	Were the economic model (including structure), study methods and analysis, and the components of the numerator and denominator displayed in a clear transparent manner?	43
13	Were the choice of economic model, main assumptions and limitations of the study stated and justified?	47
14	Did the author(s) explicitly discuss direction and magnitude of potential biases?	46
15	Were the conclusion/recommendations of the study justified and based on the study results?	41
16	Was there a statement disclosing the source of funding for the study?	42

All the studies expounded their purpose clearly, and economic evaluation was the primary objective the most included studies. Most of them calculated costs appropriately and made a straightforward description of the methodology used. Most of the studies gave details of the economic model used and of the numerator and denominator components of the ICER, and also reported incremental cost and incremental cost-effectiveness ratio (ICERs) per different natural units particularly the quality of life years (QALYs). Most of them justified their conclusions based on the results obtained. The study perspective was stated in all articles except for one study did not. Most of them provided a justification for the discount rate. Also, most of the studies disclosed their funding sources except for seven studies did not.

### Outcome and cost estimates

Most of the included studies reported incremental cost and incremental cost-effectiveness ratio (ICERs) per different natural units particularly the quality of life years (QALYs). The numerical value of outcome measures ranged from -1,623,918 to 2,297,141.53. In a study by Sawad et al., the lowest numerical value was related to the comparison of strategies 4 (symptom management (SM) and alemtuzumab) and 3 (SM and natalizumab), and the highest numerical value was related to the comparison of strategies 2 (symptom management (SM) and IFN-β-1a) and 1 (SM alone) [63]. In a study conducted in Italy, the highest value for the total lifetime cost per patient treated with IFN beta-1b—250 mcg was $1,474,840.19 [42], Table 4.

**Table 4:** Outcomes and Costs of included studies

First author’s name (Year)	Outcome Measure	Interventions	Costs	QALY/YLG		ICER		Main result
				**YLG**	**QALY**			
**Smets et al. (2023)** [53]	**QALYs**	**Ocrelizumab**	635,320.02 $	-	19.2	-		-There was no clear difference in the cost-effectiveness of sequences with ocrelizumab and ofatumumab in either first- or second-line in relapsing MS -The probability of ocrelizumab being cost-effective versus ofatumumab in first- and second-line
		**Ofatumumab**	622,623.28 $	-	18.5	-		
**Matni et al. (2022) **[54]	**QALY, LY & ICER**	**Cladribine tablets**	239,094.67 $	20.225	7.186	Reference		According to the cost-utility analysis, base case analysis, Sensitivity analysis and budget impact analysis, cladribine tablets are an economically dominant therapeutic strategy when compared to alemtuzumab, fingolimod, and natalizumab, at a threshold of 22,000 USD per QALY gained
		**Alemtuzumab**	277,825.92 $	20.225	6.947	Cladribine dominant		
		**Fingolimod**	309,969.41 $	20.225	6.150	Cladribine dominant		
		**Natalizumab**	306,363.66 $	20.225	6.546	Cladribine dominant		
**Spelman et al. (2022)** [55]	**QALY, LY & ICER**	**Natalizumab**	671,819.07 $ (Total direct cost)	20.05	7.87	Dominant		Natalizumab dominated (higher QALYs and lower costs) fingolimod in the base-case cost-effectiveness analysis (0.453 higher QALYs and £20,843 lower costs per patient)
		**Fingolimod**	702,322.97 $ (Total direct cost)	20.15	7.42	Natalizumab (Dominant)		
**Espinoza et al. (2021)** [38]	**QALY, ICER**	**Natalizumab**	227,506.54 $	-	9.519	$ 0		Compared with natalizumab, cladribine was associated with incremental costs and QALYs of US$70,989 and 1.875, respectively (incremental cost-effectiveness ratio [ICER] $37,861). Ocrelizumab was extendedly dominated by cladribine and natalizumab, and alemtuzumab was dominated by cladribine. A scenario analysis of a 10% discount did not modify the results substantially, but showed a decrease in the ICER of cladribine versus natalizumab (ICER $29,833/QALY)
		**Ocrelizumab**	251,216.33 $	-	9.912	60,328.58 $ (Extended dominated)		
		**Cladribine**	301,267.79 $	-	11.394	33,772.86 $		
		**Alemtuzumab**	305,091.44 $	-	10.786	-6288.25 $ Dominated by Cladribine		
**Bohlega et al. (2021)** [56]	**QALY, LY & ICER**	**Cladribine**	759,525.24 $	21.451	7.378	Reference		-Cladribine tablets were dominant strategy (ie, less costly and more effective) versus all the comparators - Cladribine tablets showed an 81% to 100% probability of being cost-effective at a threshold of Saudi Riyal 225 326 per quality-adjusted life-years gained against different comparators
		**Alemtuzumab**	861,168.34 $	21.451	7.134	− 13.4		
		**Dimethyl fumarate**	863,402.74 $	21.451	6.371	− 12.1		
		**Fingolimod**	894,601.38 $	21.451	6.297	− 15.6		
		**nterferon beta-1a (SC)**	774,481.67 $	21.451	5.761	− 2.3		
		**Interferon beta-1a (IM)**	793,151.61 $	21.451	6.225	− 4.3		
		**Interferon beta-1b**	843,815.62 $	21.451	7.229	− 10.6		
		**Natalizumab**	1,106,687.37 $	21.451	6.703	− 52.9		
		**Teriflunomide**	782,583.71 $	21.451	6.121	− 2.1		
**Ayati et al. (2021)** [43]	**QALY, LYG & ICER**	**Ocrelizumab**	109,029.19 $	8.525	5.459	Dominate		Ocrelizumab dominated natalizumab and was associated with cost-savings of 6971 USD, longer LYG (0.004), and higher QALYs (0.27)
		**Natalizumab**	116,145.76 $	8.521	5.192	Ocrelizumab Dominates		
**Ayati et al. (2021) **[44]	**QALY, LYG & ICER**	**Cladribine**	Total discounted cost per patient: 69,842.00 $	4.655	2.720 per patient	Dominate		Cladribine tablets dominated natalizumab and yielded 6,607 USD cost-saving and 0.003 additional QALYs per patient and also were cost-effective in Iran, with a probability of 57.5% and 58.6% at lower and higher limits of threshold, respectively
		**Natalizumab**	Total discounted cost per patient: 76,449.00 $	4.655	2.716 per patient	Cladribine Dominates		
**Lasalvia et al. (2020)** [57]	**QALY & ICER**	**Natalizumab**	75,812.51 $	-	3.01	Dominate, -2014.84 $		Natalizumab showed lower total costs (USD 80 024 vs USD 98 137) and higher QALY yield (3.01 vs 2.94) than fingolimod, dominating it (ICER = − $1861)
		**Fingolimod**	106,249.46 $	-	2.944	-		
**Poveda et al. (2021)** [39]	**QALY**	**Cladribine**	253,209.48 $ (Total cost)	-	10.39	Dominate		Cladribine tablets was the dominant treatment: lower costs (− 86,536 €) and more effective (+ 1.11 QALYs) compared to fingolimod. The probability that Cladribine Tablets was cost-effective compared to fingolimod ranged between 94.6% and 96.1% for willingness to pay from € 20,000 to € 30,000 per QALY gained
		**Fingolimod**	392,017.87 $ (Total cost)	-	9.28	Cladribine (dominate)		
**Dembek et al. (2014)** [27]	**QALY & ICER**	**Best supportive care**	496,769.72 $	-	13.07	Reference		Total QALYs gained per patient were greatest with intramuscular interferon beta-1a, followed by subcutaneous interferon beta-1a, Interferon beta-1b and Glatiramer acetate. The mean per-patient costs were lowest with intramuscular interferon beta-1a, followed by Glatiramer acetate, Interferon beta-1b, and subcutaneous interferon beta-1a. The ICERs for intramuscular interferon beta-1a was lowest at €168,629 per QALY gained
		**Intramuscular interferon beta-1a**	740,101.11 $	-	13.94	279,305.22 $		
		**Interferon beta-1b**	770,855.84 $	-	13.78	384,025.01 $		
		**Subcutaneous interferon beta-1a**	879,232.81 $	-	13.85	489,674.00 $		
		**Glatiramer acetate**	760,584.94 $	-	13.57	528,067.72 $		
**Ginestal et al. (2023)** [61]	**QALY & ICER**	**Cladribine tablets**	299,481.50 $	-	6.6577	Dominant		Cladribine tablets were the dominant treatment, with lower costs and greater effectiveness per patient, compared with dimethyl fumarate
		**Dimethyl fumarate**	411,464.19 $		6.4657	-		
**Furneri et al. (2019)** [28]	**QALY, LYs & ICER**	**Natalizumab (“escalation strategy (ESC)”)**	1,017,700.64 $ (Total cost)	20.10	11.19	Dominant		Early escalation to natalizumab is dominant vs. switching among immunomodulators, in RRMS patients who do not respond adequately to conventional immunomodulators
		**Interferons/glatiramer acetate ( “switching strategy”)**	1,045,232.01 $ (Total cost)	19.67	9.67	ESC, Dominant		
**Cortesi et al. (2022)** [60]	**QALY, LYs & ICER**	**Interferon beta-1b**	212,009.74 $	17.77	4.44	-		Compared to interferon beta-1b, siponimod seems to be cost-effective in SPMS patients and sustainable, with less than 1% overall budget increased in the next 3 years
		**Siponimod**	254,164.12 $	18.05	5.49	28.891		
**Stanisic et al. (2019)** [34]	**QALY & ICER**	Alemtuzumab	540,381.77 $	-	7.11	Dominant		Alemtuzumab yielded more QALYs, incremental QALYs, less costs compared to the other DMTs in all base-case analyses. Alemtuzumab carried the highest likelihood of being below the accepted willingness-to-pay threshold (€40,000) compared to other DMTs
		Subcutaneous IFN β-1a	546,557.15 $	-	5.49	alemtuzumab VS IFN β-1a: 6173.94 $		
		Natalizumab	657,162.64 $	-	6.08	Alemtuzumab VS natalizumab: 116,780.87 $		
		Fingolimod	617,795.17 $	-	5.75	Alemtuzumab VS fingolimod: 77,413.39 $		
**Montgomery et al. (2022)** [58]	**QALY, LYs & ICER**	**Siponimod**	481,655.67 $	16.39	3.45	-		QALYs were greater for siponimod versus all comparators. ICERs, calculated as cost per QALY, for siponimod versus natalizumab (dominant), ocrelizumab (£4,760), fingolimod (£10,033) and dimethyl fumarate (£15,837) indicated that siponimod was cost-effective at the commonly accepted willingness-to-pay threshold of £30,000/QALY
		**Natalizumab**	499,002.87 $	16.25	2.69	Dominant		
		**Ocrelizumab**	477,175.04 $	16.26	2.79	4,760		
		**Fingolimod**	472,502.15 $	16.26	2.81	10,033		
		**Dimethyl fumarate**	467,324.25 $	16.26	2.82	15,837		
		**Teriflunomide**	451,629.84 $	16.26	2.83	33,689		
**Rezaee et al. (2022)** [29]	**QALY & ICER**	**Rituximab**	5512.03 $	-	7.77	0.125, Dominant		Patients receiving rituximab had lower costs ($ 58,307.93 vs. $ 354,174.85) and more QALYs (7.77 vs. 7.65). In addition, the incidence of relapse by rituximab was lower compared to natalizumab (1.15 vs. 2.57). The scatter plots also showed that rituximab was more cost-effective for the patients in 100% of the simulations for the threshold of < $ 37,641
		**Natalizumab**	36,811.05 $	-	7.65	0, Rituximub (dominant)		
**Becker et al. (2011)** [47]	**-**	**Intramuscular interferon beta-1a**	-In the original model, costs per relapse avoided: 171,088.83 $ - In the reanalysis using the 2-year completer data, costs per relapse avoided: 94,139.24 $	-	-	-		The cost per relapse avoided for intramuscular interferon beta-1a was approximately 45% lower than in the original analysis, whereas the recreated results for the other 3 therapies differed from the original results by less than 1%
		**Subcutaneous interferon beta-1a**	-In the original model, costs per relapse avoided: 97,288.88$ - In the reanalysis using the 2-year completer data, costs per relapse avoided: 96,723.90 $	-	-	-		
		**Subcutaneous interferon beta-1b**	-In the original model, costs per relapse avoided: 105,102.03$ - In the reanalysis using the 2-year completer data, costs per relapse avoided: 104,511.70 $	-	-	-		
		**Glatiramer acetate**	-In the original model, costs per relapse avoided: 106,609.85 $ - In the reanalysis using the 2-year completer data, costs per relapse avoided: 105,954.33 $	-	-	-		
**Kantor et al. (2023)** [48]	**ICER**	**Ozanimod **(1 mg)	Total MS-Related Healthcare Costs Per Relapse Avoided: 843,684.00 $	-	-	823,168.00 $		Compared with other DMTs, treatment with ozanimod was associated with annual healthcare cost savings ranging from $2178 (vs fingolimod) to $8257 (vs interferon beta-1a 30 μg) based on a budget of 1 million USD
		**Teriflunomide (7 mg)**	491,186.00 $	-	-	480,603.00 $		
		**Teriflunomide (14 mg)**	259,369.00 $	-	-	247,052.00 $		
		**Interferon beta-1b (250 mg)**	-	-	-	294,331.00 $		
		**Interferon beta-1a (22 mcg)**	-	-	-	437,919.00 $		
		**Interferon beta-1a (30 mcg)**	843,684.00 $	-	-	823,168.00 $		
		**Interferon beta-1a (44 mcg)**	338,676.00 $	-	-	333,590.00 $		
		**Glatiramer acetate (20 mg)**	158,154.00 $	-	-	154,035.00 $		
		**Glatiramer acetate (40 mg)**	110,364.00 $	-	-	105,133.00 $		
		**Fingolimod (0.5 mg)**	72,847.00 $	-	-	72,789.00 $		
		**Dimethyl fumarate (240 mg)**	-	-	-	88,468.00 $		
**Baharnoori et al. (2022)** [13]	**QALY, Yl & ICER**	**Total cost for the first-line therapies: Ofatumumab**	603,393.83 $	28.406	9.277	-		Among first-line indicated therapies for RRMS, ofatumumab was dominant (more effective, lower costs) over teriflunomide, interferons, dimethyl fumarate, and ocrelizumab. Compared with glatiramer acetate and best supportive care, ofatumumab resulted in CERs of $24,189 Canadian dollars per QALY and $28,014/QALY, respectively. At a willingness-to-pay threshold of $50,000/QALY, ofatumumab had a 64.3% probability of being cost effective. Among second-line therapies (scenario analysis), ofatumumab dominated natalizumab and fingolimod and resulted in an ICER of $50,969 versus cladribine
		**Total cost for the first-line therapies: Ocrelizumab**	637,352.93 $	28.383	9.145	Ofatumumab dominant		
		**Total cost for the first-line therapies: Teriflunomide**	618,809.71 $	28.170	7.950	Ofatumumab dominant		
		**Total cost for the first-line therapies: Dimethyl fumarate**	626,143.68 $	28.238	8.341	Ofatumumab dominant		
		**Total cost for the first-line therapies: Glatiramer acetate**	579,403.93 $	28.190	8.056	19,643.61 $		
		**Total cost for the first-line therapies: Avonex**	625,460.71 $	28.216	8.118	Ofatumumab dominant		
		**Total cost for the first-line therapies: Rebif 22**	613,977.78 $	28.202	8.085	Ofatumumab dominant		
		**Total cost for the first-line therapies: Rebif 44**	634,898.80 $	28.178	7.994	Ofatumumab dominant		
		**Total cost for the first-line therapies: Betaseron**	617,127.87 $	28.189	8.041	Ofatumumab dominant		
		**Total cost for the first-line therapies: Extavia**	613,156.76 $	28.189	8.032	Ofatumumab dominant		
		**Total cost for the first-line therapies: Best Supportive Care**	559,939.80 $	28.073	7.367	22,749.84 $		
		**Total cost for the second-line therapies: Cladribine**	581,239.25 $	28.311	8.742	41,391.33 $		
		**Total cost for the second-line therapies: Natalizumab**	706,381.25 $	28.382	9.138	Ofatumumab dominant		
		**Total cost for the second-line therapies: Fingolimod**	627,573.76 $	28.251	8.422	Ofatumumab dominant		
**Lazzaro et al. (2022)** [40]	**QALYs, LYs & ICER**	**Teriflunomide**	- **Healthcare sector perspective:** RRMS naïve patients: 126,174.07 $ **- Societal perspective:** 152,187.82 $	**-Healthcare sector perspective:** RRMS naïve patients: 6.406 **- Societal perspective:** 6.406	**-Healthcare sector perspective:** RRMS naïve patients: 3.603 **- Societal perspective:** 3.603	-		Baseline CUA shows that teriflunomide in RRMS naïve patients was strongly dominant vs. experienced patients (healthcare sector perspective: − €1042.68 and + 0.480 QALYs; societal perspective: − €6782.81 and + 0.480 QALYs)
			- **Healthcare sector perspective:** RRMS experienced patients: 127,641.15 $ **- Societal perspective:** 161,731.42 $	**-Healthcare sector perspective:** RRMS experienced patients:6.402 **- Societal perspective:** 6.402	**-Healthcare sector perspective:** RRMS experienced patients:3.123 **- Societal perspective:** 3.123	0.480		
**Pinheiro et al. (2020)** [45]	**QALY, & ICER**	**Cladribine tablets**	332,546.67 $	-	3.42	Dominant		Cladribine tablets were associated with a delay in progression, resulting in a gain of 0.85 QALYs and a cost decrease of 25,935 €. Probabilistic sensitivity analysis resulted in a mean ICER of − 31,781 € per QALY and was dominant in 98.7% of the simulations
		**Fingolimod**	404,142.19 $	-	2.58	Cladribine, Dominant		
**Martins et al. (2023)** [33]	**QALY, LYs & ICER**	**Ocrelizumab**	For RMS: 544,039.49 $ For PPMS: 558,109.75 $	For RMS: 15.24 For PPMS: 14.13	For RMS: 3.22 For PPMS: 1.27	-		-Ocrelizumab is expected to increase (undiscounted) life expectancy of PPMS patients by 0.55 LY (25.15 vs 24.59 years) relative to BSC - Both natalizumab and ocrelizumab can reduce the number of attacks (relapses) relative to the other compared DMTs
		**BSC**	For PPMS: 451,126.90 $	For PPMS: 13.94	For PPMS: 0.47	133,729.41 $		
		**Interferon β-1a**	For RMS: 512,571.75 $	For RMS: 15.05	For RMS: 2.11	28,349.12 $		
		**Dimethyl fumarate**	For RMS: 532,077.14 $	For RMS: 15.08	For RMS: 2.29	12,862.84 $		
		**Glatiramer acetate**	For RMS: 525,298.92 $	For RMS: 15.04	For RMS: 2.02	15,616.86 $		
		**Teriflunomide**	For RMS: 514,460.90 $	For RMS: 15.06	For RMS: 2.16	27,904.81 $		
		**Fingolimod**	For RMS: 593,567.77 $	For RMS: 15.06	For RMS: 2.15	Dominant		
		**Natalizumab**	For RMS: 598,975.76 $	For RMS: 15.21	For RMS: 2.92	Dominant		
**AlRuthia et al. (2021)** [49]	**-**	**Oral agents**	10,819.76 $	-	-	Dominant		The use of orally administered agents was dominant (e.g., more effective and less costly), with average annual cost savings of USD − 4336.65 and 8.11% higher rate of effectiveness when compared with Rebif®. With regard to the use of MABs in comparison to Rebif®, MABs were associated with higher cost but a better rate of effectiveness, with an average additional annual cost of USD 1381.54 and 43.11% higher rate of effectiveness. The use of MABs in the management of RRMS among the young patient population has shown to be the most effective therapy in comparison to both IFN-based therapy (e.g., Rebif®) and orally administered agents, but with higher cost. Orally administered agents resulted in better outcomes and lower costs in comparison to IFN-based therapy
		**Interferon**	15,068.10 $	-	-	-		
		**Monoclonal antibodies (MABs)**	16,421.20 $	-	-	-		
**Versteegh et al. (2022)** [19]	**QALY & ICER**	**PEG-GLA20-OCR-CLA3.5-ALE**	672,081.80 $	-	19.59	-		Optimal lifetime health outcomes (20.24 QALYs, 6.11 relapses) were achieved with the sequence peginterferon-dimethyl fumarate-ocrelizumab-natalizumab-alemtuzumab. The most cost-effective sequence (peginterferon-glatiramer acetate-ocrelizumab-cladribine-alemtuzumab) yielded numerically worse health outcomes per patient (19.59 QALYs, 6.64 relapses), but resulted in €98 127 less costs than the most effective treatment sequence
		**PEG-DIF-OCR-CLA3.5-ALE**	676,300.27 $	-	19.65	-		
		**PEG-GLA20-CLA3.5-OCR-ALE**	654,324.96 $	-	19.29	-		
		**PEG-TER14-OCR-CLA3.5-ALE**	674,927.06 $	-	19.61	-		
		**PEG-DIF-CLA3.5-OCR-ALE**	659,500.83 $	-	19.36	-		
		**PEG-TER14-CLA3.5-OCR-ALE**	657,541.11 $	-	19.32	-		
		**IFNb250-GLA20-OCR-CLA3.5-ALE**	646,464.25 $	-	19.07	-		
		**IFNb250-DIF-OCR-CLA3.5-ALE**	482,732.38 $	-	19.13	-		
		**IFNb250-GLA20-CLA3.5-OCR-ALE**	628,417.36 $	-	18.78	-		
		**IFNb250-TER14-OCR-CLA3.5-ALE**	649,200.42 $	-	19.09	-		
**Nakhaipour et al. (2020)** [59]	**QALY & ICER**	**Fingolimod**	58,751.04 $	-	1.500	23,886		Compared with IFN β-1a, fingolimod led to an increase in QALYs with incremental costs and to an ICER of CAD 23,886/QALY over a time horizon of two years
		**IFN b-1a**	56,189.01 $	-	1.376	56,737		
**Schur et al. (2021)** [30]	**QALYs, LYs & ICER**	**Siponimod and BSC**	462,785.66 $	18.896	7.495	Dominant		In the base-case analysis, siponimod may be cost-effective for treating Swiss adult patients with SPMS with active disease
		**Interferon beta-1a and BSC**	393,591.69 $	18.412	5.905	-		
**Albahari et al. (2023)** [62]	**-**	**Rituximab**	7364.03 $	-	-	Dominant		Rituximab was more effective and less costly than natalizumab in the management of RRMS. Ocrelizumab did not seem to slow the rates of disease progression among patients previously treated with natalizumab
		**Natalizumab**	19,301.91 $	-	-	Rituximab, dominant		
		**Ocrelizumab**	35,222.92 $	-	-	-		
**Gani, et al. (2008)** [22]	**QALY & ICER**	**Natalizumab**	-	-	-	-		If UK society is willing to pay more than £8200 per QALY, or Health and Social Services are willing to pay more than £26 000 per QALY, this analysis suggests that natalizumab is likely to be a cost-effective treatment for all patients with RRMS
		**Interferon-β**	-	-	-	The ICER for natalizumab compared with interferon-β was £2300 per QALY. From a health and social care cost perspective, the ICERs were £18 700 per QALY		
		**Glatiramer acetate**	-	-	-	The ICER for natalizumab compared with glatiramer acetate was £2000 per QALY. From a health and social care cost perspective, the ICERs were £20 400 per QALY		
		**Best supportive care**	-	-	-	The ICER for natalizumab compared with best supportive care was £8200 per QALY. From a health and social care cost perspective, the ICERs were £25 500 per QALY,		
**Chilcott, et al. (2003)** [2]	**Cost per quality**	**Interferon betas**	The base case cost per quality-adjusted life-year gained by using any of the four treatments ranged from £42 000 ($66 469; &61 630) to £98 000 based on efficacy information in the public domain	-	-	-		Cost-effectiveness varied markedly between the interventions. Uncertainty around point estimates was substantial., Price was the key modifiable determinant of the cost-effectiveness of these treatments
		**Glatiramer acetate for relapsing–remitting**	Increased the cost per QALY gained around 75%	-	-			
		**Interferon betas**	The estimates with a 20-year time horizon were markedly lower, ranging from £42 000 to £98 000 per QALY gained	-	-	-		
		**Glatiramer acetate for secondary progressive multiple**	Commercial in­confidence estimates of efficacy, the most favorable estimate is £35 000 per QALY and the least favorable is £104 000 per QALY	-	-	-		
**Chevalier et al., (2016)** [17]	**QALY & ICER**	**DMF**	$ 1,191,203.33	-	5.271	-		Dimethyl fumarate can be considered a cost-effective option as it is on the efficiency frontier
		**IFN beta-1a** **44mcg**	$ 1,185,485.36	-	4.990	-		
		**IFNbeta-1a 30mcg**	$ 1,191,212.65	-	4.991	-		
		**IFN beta-1b, 250mcg**	$ 1,207,191.61	-	4.819	-		
		**Glatiramer** **Acetate**	$ 1,208,023.54	-	4.950	-		
		**Teriflunomide**	$ 1,192,521.07	-	5.047	-		
		**Fingolimod**	$ 1,267,970.65	-	5.021	-		
**Chanatittarat, et al. (2016)** [35]	**ICER**	**BSC**	BSC had the lowest cost = $235,000	-	49%	-		Compared with fingolimod and interferon treatments, BSC remains to be the most cost-effective treatment for RRMS in Thailand based on a WTP threshold of $4,500 per QALY gained
		**fingolimod**	the highest cost = $285,000	10.80	5.26 (%18)	$33,000 When compared with BSC		
		**IFNβ − 1b**	-	-	%25	$12,000 When compared with BSC		
		**IFNβ − 1a**	-	-	-	$42,000 When compared with BSC		
**Brown, et al. (2000)** [52]	**1-Disability years avoided (DYA)** **2- Cost per exacerbation avoided** **3-ICER**	**Interferon beta-1b**	1- Cost per disability year avoided before discounting is $124,892, and $181,395 after discounting at 5% 2- Total healthcare costs for all EDSS scores for Females Per person with MS: $1,976 Total healthcare costs for all EDSS scores for Males Per person with MS:$1,683	-	-	-		Using the Expanded Disability Status Scale, cost per disability year avoided due to interferon beta-1b treatment in RRMS is quite high
**Bozkaya, et al. (2017)** [23]	**ICER & EDSS**	**Natalizumab (NTZ)**	Annual Drug cost: $71,773	-	-	-		Costs ranged from $561,177 (NTZ) to $616,251 (GA). NTZ, DMF, and PEG were dominant (less costly and more effective) compared to FIN, GA, and IFN, respectively, for all ICERs
		**Fingolimod (FIN)**	Annual Drug cost: $77,922	-	-	Incremental cost NTZ vs FIN -$35,524		
		**Peginterferon beta-1a (PEG)**	Annual Drug cost: $72,072	-	-	-		
		**Subcutaneous interferon beta-1a (IFN, 44 mcg)**	Annual Drug cost: $77,797	-	-	Incremental cost PEG vs IFN-$37,790		
		**Glatiramer acetate** **(GA, 20 mg daily**	Annual Drug cost: $80,436	-	-	-		
		**Dimethyl fumarate (DMF)**	Annual Drug cost: $73,371	-	-	-		
**Alsaqa’aby et al. (2017)** [24]	**ICERs and NMB**	**Interferon 1a (Rebif 44 mcg)**	$298 892	-	9.78	-		1-None of the DMDs were found to be cost-effective in the treatment of RRMS at a WTP threshold of $100,000 in this analysis 2- Monte Carlo simulation results showed that Rebif was the most cost-effective therapy at WTP of $50 000 with 95% probability 3- Avonex reported the lowest ICER value of $337 282/QALY compared to Rebif as a common comparator 4- The NMB of oral DMDs at a WTP of $100,000 (SAR375 000) was lower than the NMB of Rebif, showing that oral DMDs were a costly option would only be cost-effective at a WTP above $300 000
		**Teriflunomide**	$360,631	-	9.72	Dominated		
		**Interferon 1a** **(Avonex 30 mcg)**	$374,502	-	10.01	$337,282		
		**Fingolimod**	$391,603	-	10.05	$347,338		
		**Dimethyl Fumarate** **(DMF)**	$426,030	-	10.02	$531,329		
**Hernandez et al. (2016)** [18]	**QALY & ICER**	**Peginterferon** **beta-1a**	-	-	Results Over 10 years, peginterferon beta-1a was dominant (i.e., more effective and less costly), with cost-savings of $22,070 and an additional 0.06 QALYs when compared with interferon beta-1a 44mcg	-		This analysis suggests that long-term treatment with peginterferon beta-1a improves clinical outcomes at reduced costs compared with interferon beta-1a 44 mcg and glatiramer acetate 20 mg and should be a valuable addition to managed care formularies for treating patients with RRMS
		**interferon beta-1a (44 mcg SC 3 times per week)**	-	-	Results Over 10 years	-		
		**glatiramer acetate (20 mg SC once daily)**	-	-	Peginterferon beta-1a was dominant (i.e., more effective and less costly), with cost-savings of $19,163 and 0.07, QALYs gained when compared with glatiramer acetate 20 mg.-	-		
**Hernandez et al. (2017)** [25]	**QALY & ICER**	**Peginterferon beta-1a**	Total cost: 106,843	-	Total QALYs (patient-caregive): 7.32	-		Long-term treatment with peginterferon beta-1a improves clinical outcomes, while its cost profile makes it either dominant or cost-effective compared with other self-injectable DMTs for the treatment of RRMS in Scotland
		**Interferon beta-1a 30 mcg**	Total cost: 113,257	-	Total QALYs (patient-caregiver): 6.88	-		
		**Interferon beta-1a 22 mcg**	Total cost: 115,614	-	Total QALYs (patient-caregiver): 6.99	-		
		**Interferon beta-1a 44 mcg**	Total cost: 112,523	-	Total QALYs (patient-caregiver): 7.01	-		
		**Interferon beta-1b**	Total cost: 110,657	-	Total QALYs (patient-caregiver): 6.88	-		
		**Glatiramer acetate 20 mg**	Total cost: 104,441	-	Total QALYs (patient-caregiver): 6.90	-		
**Sawad et al. (2017)** [63]	**QALY & ICER**	**Strategy 1: SM (symptom management)**	US$161,136.60	-	10.49	2,297,141.53 comparing Strategy 2 to Strategy 1		Strategy 1 was the cost-effective strategy for the treatment of relapsing–remitting multiple sclerosis when compared with other strategies
		**Strategy 2: SM and** **IFN-β-1a**	US$551,650.66	-	10.66			
		**Strategy 3: SM and natalizumab**	US$703,463.60	-	10.69	-1,623,918.00 comparing Strategy 4 to Strategy 3		
		**Strategy 4: SM and alemtuzumab**	US$670,985.24	-	10.71			
**Hashemi-Meshkini A, et al. (2018)** [26]	**QALY**	**PEG-interferon**	1- total discounted cost PEG-interferon: 68,688 USD 2-In each arm, cost of PEG-interferon 99% total cost	-	5709.88	1- (ICER) was estimated around 11,111 US dollars (USD) per QALY gained for the PEG-interferon vs. interferon 2- ICER (USD per QALY): cost discount rate (5%) = 12,080 3- ICER (USD per QALY): Utility discount rate (3%) = 10,208		PEG interferon beta 1 -a could be considered as a cost-effective treatment for Iranian patients with MS
		**Interferon**	1-total discounted cost in interferon arm: 59,308 USD 2- In each arm, interferon beta 1a were around and 97%total cost	-	4865.61			
**Else Michels et al. (2019)** [32]	**QALY& ICER**	**Cladribine tablets**	$ 180.67	-	9.318	Dominant		Cladribine tablets are cost-effective versus alemtuzumab and fingolimod in HAD (high disease activity) patients, and cost-effective versus natalizumab in RES (rapidly evolving severe) patients
		**Alemtuzumab**	$ 1153.24	-	9.219	Dominant		
		**Fingolimod**	$ 1397.65	-	8.333	Dominant		
		**Natalizumab**	$ 670.29	-	8.794	Dominant		
**Imani et al., (2012)** [36]	**QALY/ Incremental cost per QALY gained**	**Symptom Management**	-	-	9.081	Reference		Disease-modifying drugs (DMDs) in relapsing–remitting MS patients were associated with increased benefits compared with symptom management, albeit at higher costs. Because patients receiving Avonex incurred slightly higher QALYs than patients receiving other DMDs, treatment with Avonex dominates other DMDs in Iran
		**Avonex**	$125,280	-	9.285	$607,397		
		**Betaferon**	$280,581	-	9.284	$1,374,355		
		**Rebif**	$232,740	-	9.279	$1,166,515		
		**CinnoVex**	$50,448	-	9.130	$1,010,429		
**Janković et al., (2009)** [31]	**QALY/ Incremental cost per QALY gained/ Incremental cost per life years gained**	**Symptom** **management**	$ 321,263.12	Life years gained 16.0 ± 7.0	9.2 ± 4.2	Reference		Immunomodulatory therapy of RRMS in a Balkan country in socioeconomic transition is not cost-effective, regardless of the type of the therapy. The moderate gain in relapse-free years does not translate to gain in QALYs, probably due to adverse effects of immunomodulatory therapy
		**SC GA**	$ 566,722.58	16.4 ± 7.0	9.8 ± 4.4	1,240 ± 15,596		
		**SC IFN β-1a**	$ 924,082.67	16.4 ± 7.0	9.8 ± 4.3	4,520 ± 61,855		
		**IM IFN β-1a**	$ 920,472.98	16.4 ± 7.0	9.8 ± 4.4	4,527 ± 61,854		
		**SC IFN β-1b**	$ 855,498.41	16.4 ± 7.0	9.8 ± 4.3	4,022 ± 55,055		
**Maruszczak et al., (2015)** [41]	**QALY & ICER**	**Fingolimod**	$ 564,448.36	-	4.70	12,528		Fingolimod remains cost-effective in highly active (HA) RRMS following the introduction of DMF to the UK market, and this model supports the evidence that has led it to be the only oral DMT reimbursed for HA RRMS in England
		**dimethyl fumarate (DMF**	$ 549,139.70		3.93			
**Mantovani (2019)** [42]	**QALY, YLG ICER**	**Dimethyl fumarate**	$ 1,396,605.43	19.634	6.526	Reference		This cost-effectiveness analysis confirms that dimethyl fumarate is an optimal first-line treatment for RRMS in Italy, compared with the other first-line alternatives
		**IFN beta-1a – 22 mcg**	$ 1,418,953.20	19.533	5.786	DMF dominates		
		**IFN beta-1a – 44 mcg**	$ 1,409,201.85	19.600	6.189	DMF dominates		
		**IFN beta-1b – Betaferon**	$ 1,474,840.19	19.440	5.143	DMF dominates		
		**IFN beta-1b – Extavia**	$ 1,468,349.53	19.440	5.143	DMF dominates		
		**Glatiramer acetate – 20 mg**	$ 1,454,399.37	19.459	5.341	DMF dominates		
		**Teriflunomide – 14 mg**	$ 1,421,793.87	19.547	5.953	DMF dominates		
**Najafi et al., (2014)** [50]	**Health-related quality of life (HRQoL) & ICER**	**CinnoVex**	Annual per-patient cost: $2410	-	69.5 for physical HRQoL & 63.3 for mental HRQoL	Reference		The results showed that CinnoVex was less expensive and more effective than Avonex over the study period. This implies that CinnoVex is a dominant option and there is no need to calculate the ICER
		**Avonex**	Annual per-patient cost: $4515	-	50.9 for physicalHRQoL & 56.6 for mental HRQoL	CinnoVex dominates		
**Nuijten et al. (2002)** [46]	**QALY & ICER**	**Preventive** **treatment with interferon beta**	$ 455,373.06	-	Interferon group: 28.2	$ 106,076.04 per QALY		Preventive treatment with interferon beta in patients with multiple sclerosis may not be fully justified from a health-economic perspective, although interferon beta is associated with improved effectiveness compared with no preventive treatment
		**No preventive treatment**	$ 105,319.26	-	no-treatment group: 24.9			
**Soini et al., (2017)** [10]	**QALY & ICER**	**DMF 240 mg PO BID**	Total costs/patient, $ 523,140.50	12.098	Total QALY/patient 7.808	$ 51,149.25	$ 114,552.40	Teriflunomide was less costly, with greater QALYs, versus glatiramer acetate and the IFNs. According to Bayesian treatment ranking (BTR), teriflunomide was the first-best among the disease-modifying therapies, with potential willingness-to-pay thresholds of up to €68,000/QALY gained. In the IIA (impact investment assessment), teriflunomide was associated with the longest incremental quality-adjusted survival and time without cane use
		**Teriflunomide 14 mg once daily**	512,918.55	12.096	7.719	$ 36,570.33	vs. teriflunomide	
		**GA 20 mg SC once daily**	553,208.02	12.087	7.475	$ 377,612.44	Dominant	
		**IFN-β1a 44 mg SC TIW**	521,832.96	12.092	7.595	$ 87,610.24	Dominant	
		**IFN-β1b 250 mg SC EOD**	613,172.97	12.074	7.063	Dom	Dominant	
		**IFN-β1a 30 mg IM QW**	544,899.55	12.088	7.456	$ 370,707.19	Dominant	
		**Best supportive care (BSC)- placebo**	498,725.36	12.084	7.331	vs. BSC	$ 36,570.33	
**Su et al., (2016)** [64]	**QALY & ICER, HRQoL**	**DMF**	$243,079	12.124	5.885	- Reference		DMF can be considered a cost-effective option compared to other first-line DMTs
		**Glatiramer** **Acetate (GA)**	$219,741	12.105	5.357	$44,118		
		**Rebif (Interferon-b 1a SC)** **44 mcg**	$240,134	12.116	5.610	$10,672		
**Zhang et al., (2014)** [51]	**QALY, ICER & incremental net monetary benefit (INMB)**	**Fingolimod**	$239,947		3.69	$ 46,328	$ 36,567	Of the four DMDs, dimethyl fumarate is a dominant strategy to manage RRMS. Dimethyl fumarate dominated all other therapies over the range of willingness-to-pay (WTP) values. After dimethyl fumarate, teriflunomide was the most cost-effective therapy compared with IM IFN-b1a, with an incremental cost-effectiveness ratio of $7,115
		**Teriflunomide**	$226,085		3.68	$7,115	$ 49,780	
		**Dimethyl fumarate**	$200,145		3.72	Dominant	$ 80,611	
		**Intramuscular (IM) interferon (IFN)-b1a**	$223,606		3.34	ICER vs. IM IFN-b1a	INMB vs. IM IFN-b1a	
**Zimmermann et al., (2018)** [37]	**QALYs & ICERs**	**Ocrelizumab (for first-line treatment for RRMS)**	US$1,217,737	-	US$166,338	Dominant		Ocrelizumab was cost effective as a first-line treatment for RRMS. Alemtuzumab dominated other options for second-line treatment of RRMS
		**Alemtuzumab (for second-line treatment)**	US$580,052	-	US$648,799	Dominant		
		**Supportive care**	US$341,120	-	US$341,100	-		

### The incremental cost-effectiveness ratio of the included studies

As the outcomes and protocols of each study were too heterogeneous to allow a statistical analysis of grouped data, we presented the results using a descriptive analysis approach (Table 4). Ten studies analyzed the first line of treatment [10, 24, 27, 35, 42, 47, 48, 51, 55, 64], three studies looked at both first- and second-line treatment [13, 17, 53], and two studies focused on first-, second- and third-line treatment [19, 63]. One study looked at both first- and second-line treatment of RRMS as well as first-line treatment of PPMS [37]. One study looked at for the treatment of both RMS and PPMS [33]. Moreover, tweleve studies compared DMDs in patients with highly active RRMS (HARRMS) [22, 32, 34, 38, 39, 41, 44, 45, 54–57, 61].

### First-line medications

Dimethyl fumarate was evaluated in 3 studies [10, 42, 64], one study compared the cost-effectiveness of fingolimod, teriflunomide, dimethyl fumarate, and intramuscular interferon-b1a [51], one study assessed the cost-effectiveness of oral agents (e.g. fingolimod, teriflunomide, dimethyl fumarate) in RRMS compared to interferon-based therapy (Avonex and Rebif) [24], one study evaluated the cost-utility of MS treatments compared with best supportive care [35], one study compared the cost-effectiveness of injectable DMTs (interferon beta-1a, subcutaneous interferon beta-1a, interferon beta-1b and glatiramer acetate) [27], one study evaluated the cost-effectiveness of first-line oral DMTs (ozanimod fingolimod, dimethyl fumarate, and teriflunomide) and injectable DMTs (interferon beta-1a, interferon beta-1b, and glatiramer acetate) [48], one study estimated the cost-effectiveness of switching to natalizumab compared with switching to fingolimod with inadequate response to other DMTs [55], one study compared cost-effectiveness of intramuscular interferon beta-1a versus subcutaneous interferon beta-1a, interferon beta-1b, and glatiramer acetate [47], one study assessed the cost-effectiveness of ofatumumab [13], one study compared the cost-effectiveness of dimethyl fumarate, glatiramer acetate, interferon β-1a, interferon β-1b, peginterferon β-1a, teriflunomide, natalizumab, fingolimod, and ocrelizumab [37].

According to Mantovani et al., Su et al. and Zhang et al. [42, 51, 64] dimethyl fumarate was more cost-effective and was associated with higher QALYs and YLs as compared with IFN beta-1a – 22 &44 mcg, IFN beta-1b – 250 mcg, interferon-b 1a, interferon-b 1b, glatiramer acetate and teriflunomide, Rebif, natalizumab, fingolimod, teriflunomide, dimethyl fumarate, and intramuscular interferon-b1a. Chanatittarat et al. demonstrated although fingolimod was not the most cost-effective treatment, it was associated with higher QALYs and LYs [35]. Alsaqa’aby et al., evaluated cost-effectiveness of oral agents (fingolimod, teriflunomide, dimethyl fumarate) in RRMS compared to interferon-based therapy (Avonex and Rebif) in Saudi Arabia and showed Rebif was an optimal therapy at a WTP threshold of $100 000. They reported although Avonex had the lowest ICER value of $337 282/QALY when compared to Rebif, it was not cost-effective at acceptable universal WTP thresholds [24].

Spelman et al. showed natalizumab dominated fingolimod (higher QALYs and lower costs) for UK patients inadequately responding to first-line (interferon-based therapies, glatiramer acetate, dimethyl fumarate, and teriflunomide (BRACETD)). They also showed switching to natalizumab was associated with a significant reduction in annualized relapse rate and an increase in improvement (CDI6M) compared to switching to fingolimod [55]. According to Kantor et al. in the US, treatment with ozanimod was associated with considerable reductions in annual drug costs and total MS-related healthcare costs to avoid relapses compared with fingolimod, dimethyl fumarate, and teriflunomide, interferon beta-1a, interferon beta-1b, and glatiramer acetate. In other words, treatment with ozanimod was associated with annual healthcare cost savings ranging from $2178 (vs fingolimod) to $8257 (vs interferon beta-1a 30 μg) [48]. Russell et al. showed intramuscular interferon beta-1a was more cost-effective than subcutaneous interferon beta-1a, interferon beta-1b, and glatiramer acetate [47]. Dembek et al. showed interferon beta-1a was more cost-effective and yielded greater QALY than subcutaneous interferon beta-1a, interferon beta-1b, glatiramer acetate and best supportive care [27].

According to Zimmermann et al. ocrelizumab dominated the other DMTs (dimethyl fumarate, glatiramer acetate, interferon β-1a, interferon β-1b, peginterferon β-1a, teriflunomide, natalizumab, fingolimod) with an ICER of US$166,338/QALY compared with supportive care and can be cost-effective as a first-line treatment for RRMS with a discounted price. They also reported ocrelizumab, peginterferon β-1a, and natalizumab added more QALYs, but at higher costs than other DMTs [37]. Smets et al., in the Netherlands showed for first-line treatment although ocrelizumab did come at a higher cost than treatment with ofatumumab, it yielded more QALYs than ofatumumab, and ofatumumab was better in prevention of relapses for first- or secondline treatment [53]. According to Baharnoori et al. in Canada ofatumumab was dominant (more effective, lower costs) compared with teriflunomide, interferons, dimethyl fumarate, and ocrelizumab, and ofatumumab resulted in ICERs of $24,189 Canadian dollars per QALY and $28,014/QALY compared with glatiramer acetate and best supportive care, respectively [13].

Furneri et al. evaluated cost-effectiveness of early escalation to natalizumab vs. switching among immunomodulators, and late escalation to natalizumab, in patients with RRMS in Italy who have failed first-line treatment with either interferon beta or glatiramer acetate. They showed that early escalation to natalizumab in RRMS patients who do not respond adequately to conventional immunomodulators (interferon beta, glatiramer acetate) led to both clinical and economic benefits, compared to switching among immunomodulators (interferon beta, glatiramer acetate) [28]. In contrast, Ayati et al. in Iran demonstrated ocrelizumab was a more cost-effective option in terms of QALYs and YLg than natalizumab in patients with RRMS who failed to respond to at least one first-line DMT [43].

### Second-line medications

Smet et al. compared differences in benefits between anti-CD20 mAbs in the Netherlands from a health-economic and societal perspective. They showed although drug sequences with ocrelizumab in second-line therapy were more cost-effective (higher cost but more QALYs) than ofatumumab, this outcome was very uncertain, according to the probabilistic analysis [53].

Baharnoori et al. evaluated the cost-effectiveness of ofatumumab from a Canadian healthcare system perspective, and showed ofatumumab dominated natalizumab and fingolimod and resulted in an ICER of $50,969 versus cladribine [13]. According to Zimmermann et al. for RRMS second-line therapy, alemtuzumab dominated natalizumab, fingolimod, and ocrelizumab, and was associated with more QALYs and lower costs [37].

### Third-line medications

Sawad et al. compared four strategies; symptom management (SM) alone, SM in combination with one of the following: IFN-β-1a, natalizumab (after switching from IFN- β-1a) and alemtuzumab (after using IFN-β-1a, then switching to natalizumab) in the US. They showed although none of the DMTs were cost-effective with respect to the threshold (threshold of USD 50,000–100,000), alemtuzumab dominated over natalizumab, regardless of the WTP per QALY threshold [63]. Versteegh et al. (5W) focused on three line treatments and compared 360 treatment escalation sequences for patients with RRMS in terms of health outcomes and societal costs in the Netherlands. They showed optimal lifetime health outcomes were achieved with the sequence peginterferon, dimethyl fumarate, ocrelizumab, natalizumab and alemtuzumab. The most cost-effective sequence (peginterferon- glatiramer acetate-ocrelizumab-cladribine-alemtuzumab) yielded numerically worse health outcomes per patient but resulted in less costs than the most effective treatment sequence [19].

### DMDs in HAD RRMs patients

Eight studies evaluated the cost-effectiveness of cladribine tablets in HDA RRMs patients.

A study in Lebonan demonstrated cladribine tablets were a cost-effective (less costly and more effective in terms of QALY) and budget-saving treatment option for the treatment of HDA RMS patients when compared with alemtuzumab, fingolimod, and natalizumab [54]. Similarly, Michels et al. in the Netherlands showed treatment of RRMS with cladribine tablets was cost-effective versus alemtuzumab and fingolimod in HDA patients, and cost-effective versus natalizumab in rapidly evolving severe (RES) patients, at a threshold of €50,000/QALY gained [32]. Bohlega et al. in Saudi Arabia showed cladribine tablets as a treatment option for patients with HDA compared with alemtuzumab, dimethyl fumarate, fingolimod, interferon beta-1a (subcutaneous and intramuscular) and beta-1b, natalizumab, and teriflunomide. Their analysis demonstrated cladribine tablets as dominant strategy (less costly and more effective in terms of QALY) [56]. Poveda et al. in Spain compared the cost-effectiveness of cladribine tablets with fingolimod in patients with HDA and showed cladribine tablets were the dominant treatment (lower costs and higher QALYs) compared to fingolimod and could generate savings for the Spanish National Health System [39]. Ayati et al., in Iran compared the cost-utility of cladribine tablets in patients with HDA-RMS with natalizumab, and showed cladribine tablets dominated natalizumab with lower cost and higher QALYs per patient [44]. Pinheiro et al. assessed the cost-utility of cladribine tablets versus fingolimod in patients with highly active RRMS in Portugal. They showed cladribine tablets was less costly and more effective and also was associated with higher QALYs and a delay in progression than treatment with fingolimod [45]. Ginestal et al. evaluated the cost–effectiveness treatment of RRMS with cladribine tablets and dimethyl fumarate in Spain. They showed cladribine tablets treatment was found to be a dominant treatment and was associated with lower costs and greater QALY compared with dimethyl fumarate [61]. Conversely, a study in Chile demonstrated, that although cladribine was associated with better QALYs in HDA MS patients, it was not a cost-effective alternative compared with alemtuzumab, natalizumab, and ocrelizumab [38].

Moreover, 5 studies evaluated the cost-effectiveness of DMTs in patients with highly active RRMS [22, 41, 55, 57]. Stanisic et al. in Italy assessed the cost-effectiveness of alemtuzumab in comparison with subcutaneous IFN β-1a, natalizumab and fingolimod in management of RRMS. Thy showed alemtuzumab yielded more QALYs and less costs compared to the other DMTs, and carried the highest likelihood of being below the accepted WTP threshold (€40,000) compared to IFN β-1a, natalizumab and fingolimod. They also reported alemtuzumab can be considered as a preferable treatment option in the management of active or highly active RRMS [34]. Gani et al. in the UK compared the cost-effectivness of natalizumab with other DMTs (interferon-β, glatiramer acetate and best supportive care) and showed natalizumab was a cost-effective treatment and was associated with higher QALY for all patients with highly active RRMS (HARRMS) [22]. Spelman et al. in the UK in a comparative effectiveness analysis showed switching to natalizumab improves clinical and economic outcomes relative to switching to fingolimod in patients with HA-RRMS with inadequate response to BRACETD, and results in higher QALYs and lower costs [55]. Lasalvia et al. evaluated the cost-effectiveness of natalizumab compared with fingolimod for treating highly active RRMS patients in Colombia with failure of first-line therapy with interferons and showed natalizumab dominated fingolimod with lower costs and higher QALYs [57]. Conversely, one study in the UK demonstrated fingolimod was a cost-effective treatment and was associated with higher QALYs than dimethyl fumarate in HAD patients [41]. Cost, QALY, threshold and ICER values of all included studies are presented in Table 4.

### DMTs in patients with PPMS & SPMS

One study evaluated the cost-effectiveness of ocrelizumab versus supportive care for first-line treatment of PPMS [37]. One study evaluated the cost-effectiveness of ocrelizumab versus supportive care for the treatment of PPMS and versus interferon β-1a, dimethyl fumarate, glatiramer acetate, teriflunomide, fingolimod, and natalizumab for the treatment of RMS [33]. Three studies [30, 58, 60] evaluated the cost-effectiveness of siponimod versus other DMTs in patients with SPMS.

Montgomery et al., in the UK evaluated the cost-effectiveness of oral siponimod versus continued oral or infused RRMS DMTs (natalizumab, ocrelizumab, fingolimod, dimethyl fumarate, teriflunomide) for patients with active SPMS. They showed siponimod was more cost-effective, yielded greater QALYs and offered a clinically beneficial treatment approach compared with the continuation of oral or infused RRMS DMTs [58]. Schur et al. evaluate the cost-effectiveness and budget impact of siponimod compared to interferon beta-1a for adult patients with SPMS with active disease. They showed siponimod may be cost-effective and yeilds more QALYs and YLs for treating Swiss adult patients with SPMS with active disease [30]. Cortesi et al. estimated the siponimod cost-effectiveness profile and its relative budget impact compared with interferon beta-1b for patients with SPMS. They showed siponimod resulted in the most effective treatment (more QALY) but also more expensive compared with interferon beta-1b [60]. Zimmermann et al. in the US demonstrated, for PPMS, ocrelizumab had an ICER of US$648,799/QALY compared with supportive care but was not cost-effective for PPMS [37]. A study by Martins et al. in Portugal demonstrated ocrelizumab could provide important health benefits as a therapy for both RMS and PPMS. Ocrelizumab was among the most effective treatment options for RMS patients compared with other DMTs and compared with BSC for PPMS patients and yielded more LYs and QALYs for RMS and PPMS patients [33].

## Discussion

We systematically reviewed the literature with the objective of analyzing recent published evidence on cost-utility and cost-effectiveness of DMDs for RRMS. To the best of our knowledge it is the first systematic review to examine the cost-ctility and cost-cffectiveness of DMDs for RRMS.

In this review ICER values exhibited a broad variability, even within one same treatment and using the same control medication. This variability can be due to the parameters selected to develop the pharmacoeconomic model, and /or the WTP per QALY threshold established.

Our results showed that the most important injectable DMDs for RRMS were interferon beta-1a (Avonex and Rebif) and beta-1b (Betaferon and Extavia), peginterferon beta-1a, intramuscular interferon beta-1a, glatiramer acetate (Copaxone), and ofatumumab.

Concerning interferon Beta, studies showed that interferon Beta (e.g. interferon beta-1b) can reduce reduce lifetime disability years by 10% [52] and is associated with an improved effectiveness compared with preventive treatment [46]. Additionally, the cost-effectivness of peginterferon beta-1a was studied in the US and Iran [18, 25, 26]. They demonstrated peginterferon beta-1a was a cost-effective strategy and was associated with lower cost and more QALY compared with interferon beta-1a, interferon beta-1b and glatiramer acetate in the treatment of RRMS.

In this review, the most important oral DMDs for RRMS were found to be teriflunomide (Aubagio), monomethyl fumarate (Tecfidera), fingolimod, cladribine, siponimod, ponesimod, DMF, diroximel fumarate, ozanimod, and cladribine tablets. The most important intravenous infusions DMDs of RRMS were alemtuzumab, mitoxantrone, ocrelizumab, natalizumab, and rituximab.

Ten studies [13, 19, 23, 33, 34, 37, 49, 53, 56, 64] evaluated the cost-effectiveness of all three forms of DMDs for RRMS, the oral DMDs. The results varied between studies. The difference in the results can attributed to several factors such as the patients selection criteria, age groups studied, medications studied, availability of drugs in each country, treatment line, setting, disease severity, demographic and socio-economic determinants measured. Eleven studies [10, 17, 24, 30, 35, 42, 48, 51, 59, 60, 64] evaluated the cost-effectiveness of injectable and oral DMDs for RRMS and reported oral drugs were more cost-effective than injectable drugs. Likewise, eight studies [32, 38, 40, 44, 54, 55, 57, 58] evaluated the cost-effectiveness of oral and intravenous infusions DMDs of RRMS, of which 5 studies reported oral drugs were more cost-effective than intravenous infusions DMDs. In general, oral medications are preferred by patients to other forms of medication due to their being non-invasive nature.

In two studies, the cost-effectiveness of injectable drugs and intravenous infusions was analyzed by symptom management. In both studies, symptom management was more cost-effective and was associated with higher QALYs and YLs when compared with IFN-β-1a, natalizumab, alemtuzumab, glatiramer acetate subcutaneous, and intramuscular interferon β-1b. This can be attributed to the lower cost of drugs and equipment [31, 63].

In our review, for the first-line treatment, dimethyl fumarate [42, 51, 64], natalizumab [55], ozanimod [48], interferon beta-1a [27], ocrelizumab [37, 43, 53], ofatumumab [13] and teriflunomide [10] were found to be more cost-effective and was associated with higher QALY. Studies in this review suggested, natalizumab was a dominant option for HDA RRMS and RRMS patients who failed first-line treatment with either fingolimod or interferons/glatiramer acetate [28, 57].

For the second-line treatment, ofatumumab [13] and alemtuzumab [37] were found to be more cost-effective and yielded more QALY. Smets et al. showed although treatment with ocrelizumab was associated with higher cost than that of ofatumumab, it yielded more QALY than ofatumumab [53].

Of 8 studies that evaluated the cost-effectiveness of cladribine tablets in HDA patients, 7 studies demonstrated cladribine tablets were a cost-effective option (less costly and greater QALY) compared with alemtuzumab, fingolimod, natalizumab, dimethyl fumarate, interferon beta-1a (subcutaneous and intramuscular), beta-1b, natalizumab, and teriflunomide in HDA patients [32, 39, 44, 45, 54, 56, 61]. Previous systematic reviews and meta-analyses also demonstrated that cladribine tablets can be an effective and safe drug and an alternative to other DMTs in achieving better treatment for RRMS, active RRMS and for a subgroup with high disease activity (HRA + DAT) populations [65, 66]. This can be due to the oral posology of cladribine tablets where the treatment effect is expected to last for up to 4 years with only 2 years of treatment [67]. This drug has no costs of administration and lower monitoring costs compared to other drugs. Also, induction therapies, such as cladribine have low discontinuation rates owing to the prearranged schedule for treatment administration [13].

Considering DMTs in HAD patients, 3 studies demonstrated natalizumab was a cost-effective treatment and was associated with higher QALY as compared with interferon-β, glatiramer acetate, best supportive care and fingolimod [22, 55, 57]. It seems natalizumab can be considered as a cost-effective treatment in HDA patients.

Considering the cost-effctivness of siponimod versus other DMTs in patients with SPMS, studies conducted in the UK, Switzerland and Italy demonstrated siponimod was more cost-effective and yielded greater QALYs [30, 58, 60] and YLs [30] for treating patients with SPMS with active disease when compared with natalizumab, ocrelizumab, fingolimod, dimethyl fumarate, teriflunomide [58], interferon beta-1a [30] and interferon beta-1b [60]. Moreover, studies in this review demonstrated ocrelizumab can provide important health benefits as a therapy for RMS and PPMS patients compared with supportive care, yielding more LYs and QALYs [33, 37]. A previous systematic review and network meta-analysis compared ocrelizumab with other treatments for RMS and demonstrated the efficacy and safety of ocrelizumab in a direct comparison with interferon β-1a 44 μg (Rebif 44 mg) [68]. This medication was approved by the US Food and Drug Administration in March 2017 and by the European Medicines Agency in January 2018 for the treatment of RMS and PPMS [68].

In this review, 3 studies evaluated the cost-effectiveness of rituximab and showed RRMS patients receiving rituximab had lower costs and more QALYs when compared with natalizumab [29, 62]. Smet et al. in the Netherlands suggested rituximab would already be the most cost-effective anti-CD20 mAb if its efficacy on 6-month CDP is comparable to traditional first-line therapies such as interferon-beta but there are no accurate estimates of rituximab’s effect on disability progression [53]. Although rituximab has not yet been approved by the United States Food and Drug Administration (USFDA) for treating MS, it has been used extensively as an off-label medication for MS control and management. Moreover, rituximab has shown more efficacy in managing RRMS when compared with fingolimod and a better safety profile than natalizumab [69, 70]. Nonetheless, some studies have shown that the use of rituximab, natalizumab, ocrelizumab, interferons, or other injectable DMTs is associated with higher rates of nonadherence in MS managment among patients, especially those with chronic health conditions [71, 72].

## Limitations

Our review has some limitations. First, our review was limited to English and Persian language publications, and there is a chance of publication bias. So future reviews should include additional languages, if feasible. Second, several studies in this review received funding from pharmaceutical or biotechnology companies. Industry sponsorship can be a source of bias as they may support a particular agenda and be influential at multiple stages of research design and implementation and influence the choice of research priorities [73]. Third, the included studies were from different countries which have varied healthcare systems which effects the overall bias of the review.

## Conclusions

We found that, of the evaluated DMTs, cladribine tablets and natalizumab were the optimal choices for patients with highly active RRMS. Siponimod was also found to be a cost-effective option for patients patients with SPMS. Among the drug strategies with different prescribing methods, oral DMDs for RRMS should be preferred to injectable drugs and intravenous infusions for various reasons such as their non-invasiveness and greater convenience for patients, and lower cost. This review showed that care-oriented strategies such as BSC and SM strategies should be preferred to drug strategies and be considered a valuable early treatment option for patients with RRMS.

Of note, the outcomes of a cost-effectiveness analysis frequently exhibit country-specific characteristics since treatment and healthcare costs data can diverge substantially across nations. Moreover, it is noteworthy that incremental costs and QALYs may vary between different settings, even if the same fundamental modeling approach is employed. Although studies show MS is a costly disease, cost estimates vary between nations. Therefore, health policy makers, neurologists, and other involved parties should base their decisions on local findings with regards to the financial burden caused by MS and the cost-effectiveness of DMTs.

### Supplementary Information

**Additional file 1.** Search Strategy.

## Data Availability

All data generated or analyzed during this study are included in this published article.
