# Supplementary material for: Cost-utility and cost-effectiveness analysis of disease-modifying drugs of relapsing–remitting multiple sclerosis: a systematic review
Source: Health Econ Rev. 2024 Feb 16;14:12. doi: 10.1186/s13561-024-00478-7 (PMC10870486; doi:10.1186/s13561-024-00478-7)
Supplement: Supplementary file 1 — Additional file 1. Search Strategy. [file 13561_2024_478_MOESM1_ESM.doc]

**Additional File 1**

Search Strategy:

**((((((((((((((((((((((((interferon beta-1b[MeSH Terms]) OR (peg interferone[Title/Abstract])) OR (plegridy[Title/Abstract])) OR (interferon beta-1a[MeSH Terms])) OR (glatiramer acetate[MeSH Terms])) OR (teriflunomide[Title/Abstract])) OR (Aubagio[Title/Abstract])) OR (Dimethyl Fumarate[MeSH Terms])) OR (fingolimod[MeSH Terms])) OR (mayzent[Title/Abstract])) OR (siponimod[Title/Abstract])) OR (ponesimod[Title/Abstract])) OR (diroximel fumarate[Title/Abstract])) OR (ozanimod[Title/Abstract])) OR (zeposia[Title/Abstract])) OR (alemtuzumab[MeSH Terms])) OR (ocrelizumab[Title/Abstract])) OR (ocrevus[Title/Abstract])) OR (natalizumab[MeSH Terms])) OR (pimozide[MeSH Terms])) OR (disease-modifying drugs[Title/Abstract])) OR (Ofatumumab[Title/Abstract])) OR (DMDs[Title/Abstract])) AND ((((Cost-Utility Analysis[Title/Abstract]) OR (Cost-Effectiveness[MeSH Terms])) OR (Cost Minimization Analysis[Title/Abstract])) OR (Economic Evaluation[Title/Abstract]))) AND ((((((((((((Primary-Progressive MS[Title/Abstract]) OR (PPMS[Title/Abstract])) OR (Multiple sclerosis[MeSH Terms])) OR (Encephalomyelitis disseminate[Title/Abstract])) OR (MS[Title/Abstract])) OR (Relapsing-Remitting MS[Title/Abstract])) OR (RRMS[Title/Abstract])) OR (Clinically isolated syndrome[Title/Abstract])) OR (CIS[Title/Abstract])) OR (Secondary-Progressive MS[Title/Abstract])) OR (SPMS[Title/Abstract])) OR (Progressive-Relapsing MS[Title/Abstract]))**
